# A Pilot Randomized Controlled Trial and Multi-Omics Analysis of Electrolysed Alkaline Water: Impacts on Gut Microbiota and Metabolic Signatures in Hyperuricemia

**DOI:** 10.3390/nu18010107

**Published:** 2025-12-28

**Authors:** Qisijing Liu, Wentao Gu, Juan Ma, Jin Wang, Miao Yu, Min Xu, Shuo Wang

**Affiliations:** Tianjin Key Laboratory of Food Science and Health, School of Medicine, Nankai University, Tianjin 300071, China; liuqisijing@126.com (Q.L.);

**Keywords:** electrolyzed alkaline water, hyperuricemia, uric acid, gut microbiota, metabolomics, randomized controlled trial

## Abstract

**Background/Objectives**: Hyperuricemia (HUA) is the second most common metabolic disease in China (24.5% in males, 3.6% in females), which can induce multiple complications such as gout and diabetes. Existing urate-lowering drugs have significant hepatorenal toxicity, necessitating safe lifestyle interventions. Electrolyzed alkaline water (EAW) as daily drinking water has shown preliminary effectiveness, but it lacks randomized controlled evidence and mechanistic studies at the microbiome–metabolome interface. **Methods**: We conducted a 12-week randomized controlled trial in 40 adults aged 18–65 years with elevated serum uric acid (SUA). Participants consumed either 1.5 L/day of EAW (pH 8.5–9.5) or purified water (pH 7.0). Clinical indicators, quality of life (SF-36), gut microbiota, and gut metabolomics were comprehensively assessed to evaluate intervention efficacy and explore potential mechanisms. **Results**: After 12 weeks, the EAW group exhibited a larger reduction in serum uric acid than the control group, along with improvements in selected physical health-related quality-of-life measures. Modest differences in gut microbial composition were observed between groups. Metabolomic analyses identified group-level differences in metabolites enriched in pathways related to purine metabolism and other urate-associated metabolic processes. **Conclusions**: This pilot randomized controlled trial suggests that consumption of EAW is associated with a modest reduction in serum uric acid. Exploratory multi-omics analyses indicate concurrent changes in gut microbiota and metabolic profiles. These findings support further investigation of electrolyzed alkaline water as a potential adjunctive, non-pharmacological option for hyperuricemia in larger and longer-term studies. **Ethics:** This trial was registered with the Chinese Clinical Trial Registry under the identifier ChiCTR2500100190. Ethical approval for the present study was granted by the Nankai University Institutional Review Board (NKUIRB2025001, 23 January 2025).

## 1. Introduction

Hyperuricemia (HUA), characterized by elevated serum uric acid (SUA) levels, has emerged as a prevalent metabolic disorder worldwide, closely paralleling the rise in dietary westernization and sedentary lifestyles. In China, epidemiological data indicate that HUA affects approximately 24.5% of men and 3.6% of women, making it the second most common metabolic condition after diabetes [1]. Beyond its role in precipitating gout and joint inflammation, persistent HUA is inextricably linked to systemic metabolic disturbances, including insulin resistance, hypertension, and chronic kidney disease [2,3,4,5,6].

Current management strategies primarily rely on pharmacologic agents such as allopurinol and febuxostat to inhibit uric acid synthesis, or uricosurics to enhance excretion [7,8]. However, their long-term use is often limited by adverse effects, including gastrointestinal toxicity, tolerance, allopurinol hypersensitivity syndrome, nephrotoxicity, hepatic and renal toxicity and hypersensitivity reactions [9,10,11]. Nonsteroidal anti-inflammatory drugs are commonly used to manage acute gout flares but do not address hyperuricemia itself and have been reported to disrupt gut microbial homeostasis [12,13]. These limitations highlight the need for safe, accessible, and sustainable non-pharmacological approaches to support uric acid management.

Hydration is a fundamental determinant of metabolic health, yet the specific impact of water quality on uric acid regulation remains under-explored. Electrolyzed alkaline water (EAW)—produced via electrolysis to possess an alkaline pH and a negative oxidation-reduction potential (ORP)—has been proposed as a functional beverage. While some preliminary studies suggest that EAW may modulate metabolic parameters and antioxidant status [14,15,16,17], evidence for urate-lowering effects is largely derived from animal studies, and corresponding data from well-controlled human studies are currently lacking [18]. EAW differs from conventional alkaline water not only in pH but also in redox potential, ionic composition, and, in some cases, the presence of dissolved molecular hydrogen. Importantly, no randomized controlled trials to date have systematically examined whether EAW consumption can influence serum uric acid levels while simultaneously assessing gut microbiome and metabolic changes in humans.

Recent research has increasingly recognized the gut microbiota as a critical regulator of uric acid homeostasis. Approximately 25% of uric acid is excreted into the intestine, where microbial metabolism contributes to purine degradation and extrarenal urate elimination [19,20]. Individuals with hyperuricemia or gout commonly exhibit gut microbial dysbiosis, including reduced short-chain fatty acid–producing bacteria and enrichment of pro-inflammatory taxa, which has been associated with impaired gut barrier function and systemic inflammation [9,12,21]. Beyond direct urate metabolism, the gut microbiota interacts with host pathways relevant to uric acid regulation, including purine and fructose metabolism, while bile acid–related signalling pathways may indirectly influence metabolic and inflammatory homeostasis [22,23,24,25].

Therefore, we conducted a 12-week randomized controlled trial comparing electrolyzed alkaline water (pH 8.5–9.5) with purified neutral water (pH 7.0) in adults with elevated serum uric acid. We hypothesized that EAW consumption would modestly reduce serum uric acid levels compared with neutral water and that this effect, if present, would be associated with changes in gut microbial composition and purine-related metabolic profiles rather than direct gastrointestinal alkalinization. By integrating clinical endpoints with microbiome and metabolomics analyses, this study aimed to explore whether EAW represents a feasible adjunctive lifestyle intervention for hyperuricemia and to clarify potential microbiota-related mechanisms.

## 2. Materials and Methods

### 2.1. Study Design and Objectives

This study was designed as a 12-week, randomized, double-blind, parallel-controlled clinical trial to investigate the effects of EAW consumption on serum uric acid (SUA) levels and related metabolic outcomes in adults with hyperuricemia (HUA).

### 2.2. Participants

Eligible participants were adults aged 18 to 65 years diagnosed with asymptomatic hyperuricemia under a regular purine diet. Enrollment criteria required fasting SUA concentrations exceeding 420 μmol/L but not exceeding 540 μmol/L in men and postmenopausal women, or exceeding 360 μmol/L but not exceeding 540 μmol/L in premenopausal women. All participants were required to have a body mass index (BMI) between 18.5 and 29.9 kg/m^2^, to be free of urate-lowering medication or supplements within the past 30 days, and to voluntarily provide written informed consent. Baseline demographic and health information, including age, sex, BMI, medical history, and current medication use, were documented for each participant prior to enrollment.

Participants were excluded if they had a diagnosis of gout or any major comorbidities that could interfere with uric acid metabolism or study safety. Specifically, exclusion criteria included: cerebrovascular disease, coronary heart disease, heart failure, uric acid nephrolithiasis, hepatic dysfunction (alanine aminotransferase [ALT] or aspartate aminotransferase [AST] levels ≥ 2.5 times the upper limit of normal), renal impairment (estimated glomerular filtration rate [eGFR] < 45 mL/min/1.73 m^2^ or ≥CKD stage 2), or secondary hyperuricemia due to hematologic disease (e.g., leukemia), malignancy (e.g., multiple myeloma), liver cirrhosis, or drug-induced causes. Individuals who had used loop or thiazide diuretics, corticosteroids, tamoxifen, or other agents that might alter SUA or body weight within 14 days prior to screening were also excluded.

Further exclusion criteria included the presence of severe cardiovascular, hepatic, renal, hematologic, or psychiatric disorders; pregnancy, lactation, or plans for conception; known allergies to any component of the test product; and habitual excessive alcohol consumption (>3 units per day). Participants receiving any urate-lowering therapy during the intervention were withdrawn. In addition, individuals with poor hydration habits—defined as consuming less than 1000 mL of plain water per day or more than 500 mL of coffee, tea, or sugar-sweetened beverages—were excluded to ensure consistent fluid intake across participants.

All participants provided written informed consent prior to their enrollment in the study. This trial was registered with the Chinese Clinical Trial Registry under the identifier ChiCTR2500100190. Ethical approval for the present study was granted by the Nankai University Institutional Review Board (NKUIRB2025001, 23 January 2025).

### 2.3. Sample Size and Randomisation

A total of 100 individuals were screened, and 40 participants who met the eligibility criteria and demonstrated good compliance during the pre-test phase were enrolled. Participants were randomly assigned in a 1:1 ratio into the EAW group or control group (20 participants in each group). Randomisation and blinding were conducted using a computer-generated allocation sequence. Both participants and investigators were blinded to group assignment.

### 2.4. Intervention

Participants in the intervention group consumed 1.5 L/day of EAW (pH 8.5–9.5), while the control group received 1.5 L/day of purified neutral water (pH 7.0). The intervention period lasted 12 weeks. EAW is produced from raw water through a series of controlled treatment steps. Briefly, the raw water is first subjected to pretreatment, followed by membrane filtration and electrolysis, during which EAW with specific physicochemical properties is generated.

To ensure the double-blind nature of the study, both the EAW and the control water were packaged in identical containers. The two types of water were indistinguishable in appearance, texture and packaging, and neither possessed a distinct taste or odour that could reveal their identity. Each container was labelled only with a specific identification code. Both the participants and the investigators involved in data collection and outcome assessment remained blinded to the group assignments throughout the intervention period. The blinding code was revealed only after the completion of the statistical analysis.

#### 2.4.1. Run-In Screening

All eligible participants underwent physical examination and laboratory testing, including measurements of height, weight, SUA, ALT, AST, and serum creatinine. From the initial screening pool, 40 participants were selected based on inclusion and exclusion criteria.

Pre-trial Run-in: Before the official intervention, a 5-day compliance test was conducted using the control product (purified water). Participants were instructed to consume the assigned amount daily and to record adherence by photographing labelled bottles. Compliance was calculated as:

Compliance = [(Prescribed dose − Missed dose)/Prescribed dose] × 100%

Participants with ≥75% compliance were deemed eligible. Those with poor tolerance or adverse reactions were excluded. A 7-day washout period followed the compliance phase, after which the 12-week formal trial was initiated.

#### 2.4.2. Intervention Period and Monitoring

Participants visited the study centre at baseline (week 0) and at weeks 4, 8, and 12 for follow-up assessments, bottle returns, and product replenishment. They were instructed to maintain their habitual diet, physical activity, and lifestyle throughout the trial. Water intake and adherence were monitored using a mobile application. Participants uploaded photographs of daily consumption (bottle labelled with date). If adherence dropped below 5 days per week or if >3 days of consumption were missed, research staff contacted participants via phone to verify reasons and document deviations. Participants with poor adherence or protocol violations were withdrawn and classified as dropouts.

### 2.5. Outcome Measures

Study outcomes were assessed at four predefined time points: baseline, week 4, week 8, and week 12 of the intervention period. The primary outcome was the change in serum uric acid concentration, which served as the principal indicator of uric acid metabolism and excretion efficiency. The secondary outcomes included multiple clinical, metabolic, and patient-reported parameters to provide a comprehensive evaluation of the intervention effects. Health-related quality of life was evaluated using the 36-Item Short Form Survey (SF-36), with emphasis on the physical functioning, bodily pain, and physical component summary domains.

During the 12-week intervention period, participants were required to complete a 3-day food diary every 2 weeks, documenting all consumed items (including meals, snacks, beverages, and the like), covering 2 weekdays and 1 weekend day. Nutrient intake data derived from these food records were computed based on the nutritional content specifications provided in China Food Composition Tables: Standard Edition (6th Edition, Volume 1) and China Food Composition Tables (6th Edition, Volume 2) [26].

Adverse events during the entire study were recorded in detail, including onset, symptoms, severity, and duration.

### 2.6. Clinical Biochemical Indices

Comprehensive biochemical assessments were performed at baseline, week 4, week 8, and week 12 to evaluate hepatic, renal, lipid, and glucose–insulin metabolic functions. Serum lipid profiles included total cholesterol (TC), triglycerides (TG), high-density lipoprotein cholesterol (HDL-C), low-density lipoprotein cholesterol (LDL-C), and free fatty acids (FFA). Liver function was assessed by alanine aminotransferase (ALT), aspartate aminotransferase (AST), the AST/ALT ratio, total protein (TP), albumin (ALB), alkaline phosphatase (ALP), total bile acid (TBA), and bilirubin fractions (total, direct, and indirect bilirubin). Renal function indicators comprised blood urea nitrogen (BUN), serum creatinine (Cr), and estimated glomerular filtration rate (eGFR) calculated using the CKD-EPI equation.

In addition, fasting plasma glucose, fasting insulin, and homeostasis model assessment of insulin resistance (HOMA-IR) were measured to assess glucose–insulin metabolism. Urinary uric acid, allantoin, and routine urinalysis (including glucose and ketone bodies) were analyzed concurrently to evaluate uric acid excretion and renal handling. All samples were collected after an overnight fast and analyzed in a certified clinical laboratory using standardized automated biochemical assays.

### 2.7. Fecal Sample Processing and Gut-Related Multi-Omics Analyses

Finally, to explore mechanistic pathways, multi-omics analyses were conducted. Gut microbiota composition was profiled using 16S rRNA sequencing, and untargeted serum metabolomics was performed via liquid chromatography–mass spectrometry (LC–MS) at baseline and week 12. These datasets were used to explore microbe–metabolite interactions and potential biological pathways mediating the urate-lowering effects of alkaline water.

#### 2.7.1. Fecal Samples Collection

Fecal samples were collected from participants at baseline and after 12 weeks of intervention. Upon collection, samples were transported to the research centre in insulated containers with ice packs to maintain low-temperature conditions. Upon arrival at the centre, all samples were immediately stored at −80 °C in a freezer for subsequent analysis.

#### 2.7.2. 16S rRNA Gene Sequencing

Total genomic DNA was isolated from the samples using the cetyltrimethylammonium bromide (CTAB) protocol. The concentration and purity of the extracted DNA were evaluated via 1% agarose gel electrophoresis. Based on the measured concentration, the DNA samples were adjusted to a final concentration of 1 ng/µL using sterile distilled water. Specific regions of the 16S rRNA genes (16S V3-V4) were amplified with barcoded specific primers. Each PCR reaction was performed in a 15 µL volume containing Phusion^®^ High-Fidelity PCR Master Mix (New England Biolabs, Ipswich, MA, USA), two µM of both forward and reverse primers, and approximately 10 ng of template DNA. Sequencing libraries were constructed using the TruSeq^®^ DNA PCR-Free Sample Preparation Kit (Illumina, San Diego, CA, USA) according to the manufacturer’s instructions, with index codes incorporated during the process. Library quality was assessed using a Qubit^®^ 2.0 Fluorometer (Thermo Scientific, Carlsbad, CA, USA) and an Agilent Bioanalyzer 2100 system (Agilent Technologies, Palo Alto, CA, USA). Finally, the libraries were sequenced on an Illumina NovaSeq platform (Illumina, San Diego, CA, USA), generating 250 bp paired-end reads.

#### 2.7.3. UPLC-MS/MS Analysis of Metabolites

For fecal nuntargeted metabolomics detection, the instruments used included a Q Exactive™ HF/Q Exactive™ HF-X mass spectrometer (Thermo Fisher, Bremen, Germany), a Vanquish UHPLC chromatograph (Thermo Fisher, Bremen, Germany), and a Hypersil Gold column (C18, 100 × 2.1 mm, 1.9 μm, Thermo Fisher, Waltham, MA, USA); the chromatographic conditions were set as follows: column temperature at 40 °C, flow rate at 0.2 mL/min, mobile phase A as 0.1% formic acid and mobile phase B as methanol. The raw data were imported into CD 3.3 software for processing: each metabolite was first screened by retention time and mass-to-charge ratio, peak area was calibrated with the first QC sample for more accurate identification, then peak extraction (with five ppm mass deviation, 30% signal intensity deviation, minimum signal intensity and adduct ions set) and quantification were conducted, target ions were integrated, molecular formulas were predicted via molecular and fragment ions and compared with mzCloud, mzVault and Masslist databases, background ions were removed using blank samples, original quantitative results were standardized by (sample metabolite’s original value)/(sample’s total metabolite value/QC1’s total metabolite value) to get relative peak areas, compounds with >30% CV of relative peak areas in QC samples were excluded, and finally metabolite identification and relative quantification results were obtained.

### 2.8. Statistical Analysis

#### 2.8.1. Biochemical Measurement and Questionnaire

Continuous variables will be expressed as mean ± SD or median (IQR), and categorical variables as frequencies (%). Between-group differences will be analyzed using independent *t*-tests or Mann–Whitney U tests, and within-group changes will be analyzed using paired *t*-tests or Wilcoxon signed-rank tests, as appropriate. To evaluate longitudinal changes and group-by-time interactions, a linear mixed-effects model (LMM) was employed. In the model, time (week), group (treatment vs. control), and the interaction term time × group were specified as fixed effects to examine differential trends in outcome measures over time between groups. The model was further adjusted for potential confounding variables, including age, sex, body mass index (BMI), baseline dietary purine intake. For the biomarker of uric acid in blood and urine, in addition to the aforementioned confounding factors, we further adjusted for the baseline levels of SUA or urinary uric acid (UUA). Participant ID was included as a random effect to account for individual baseline variability and the non-independence of repeated measures within subjects. The mixed-effects approach allows simultaneous modelling of fixed and random components and provides robust estimation in the presence of missing data under the assumption of missing-at-random (MAR). Model parameters were estimated using the maximum likelihood (ML) method. If not specifically mentioned, all statistical analyses were performed using Stata version 18.0 (StataCorp, College Station, TX, USA), and two-sided *p* values less than 0.05 were considered statistically significant.

#### 2.8.2. Gut Microbiota Analysis

Sequence analysis of operational taxonomic units (OTUs) was conducted using Uparse software (Uparse v7.0.1001, available at http://drive5.com/uparse/, accessed on 1 August 2025). For each representative sequence, taxonomic annotations were assigned using the Silva Database (http://www.arb-silva.de/, accessed on 1 August 2025) based on the Mothur algorithm. Raw sequencing data were subjected to quality control to remove low-quality and chimeric reads. Paired-end reads were merged and filtered to generate clean data, which were subsequently denoised using the DADA2 algorithm to infer amplicon sequence variants (ASVs). Taxonomic annotation was performed based on representative ASV sequences, yielding taxonomic assignments and relative abundance profiles at different taxonomic levels.

To assess the diversity, richness, and evenness of microbial communities within samples, alpha diversity was computed using seven indices in QIIME2. β-diversity analysis was performed to evaluate inter-sample differences in species complexity, using weighted and unweighted UniFrac distances in QIIME2. Principal coordinate analysis (PCoA) results were visualized using the ade4 and ggplot2 packages in R software (Version 4.0.3). Venn diagrams were generated to visually exhibit shared and unique OTU information among different samples or groups, utilizing the VennDiagram function in R and the SVG function in Perl, respectively. A set of statistical analyses, including *t*-tests and linear discriminant analysis effect size (LEfSe), was applied to identify variations in community structure. For differential abundance analyses, multiple testing was controlled using the Benjamini–Hochberg false discovery rate (FDR) correction.

#### 2.8.3. Gut Metabolomics Analysis

Raw LC-MS data were processed using the XCMS package for peak detection, retention time alignment, and peak quantification. Metabolite annotation was performed by matching accurate mass, isotope patterns, and fragmentation spectra against high-quality MS/MS databases. To ensure data robustness, only metabolites with a coefficient of variation (CV) < 30% in quality control (QC) samples were retained for downstream analyses. Identified metabolites were annotated using KEGG (https://www.genome.jp/kegg/pathway.html (accessed on 18 December 2025)) databases. For multivariate analysis, data were transformed via metaX, followed by Principal Component Analysis (PCA) and Partial least squares discriminant analysis (PLS-DA) to obtain VIP values for each metabolite. PLS-DA was employed as a supervised multivariate method to explore group separation based on metabolic profiles. Model performance was evaluated using k-fold cross-validation (7-fold cross-validation when the number of biological replicates was sufficient; otherwise, k = 2*n*, where *n* represents the number of replicates), with R^2^ and Q^2^ values used to assess goodness of fit and predictive ability. To evaluate potential overfitting, permutation testing (200 permutations) was conducted by randomly shuffling class labels; models were considered valid when the Q^2^ regression line intercept was below zero and R^2^ exceeded Q^2^. Univariate analysis involved a *t*-test for intergroup *p*-values and fold change (FC) calculation. Differential metabolites were screened using default criteria: VIP > 1, *p* < 0.05, and FC ≥ 2 or ≤0.5. Volcano plots (R package ggplot2) integrated VIP, log_2_ (FC), and −log_10_ (*p*-value) to screen target metabolites. Bubble plots (ggplot2) combined with KEGG were used for pathway analysis. Pathway enrichment analyses were performed using a hypergeometric test, and multiple testing was adjusted using the Benjamini–Hochberg FDR method.

## 3. Results

### 3.1. Participants Characteristics

**Figure 1:**
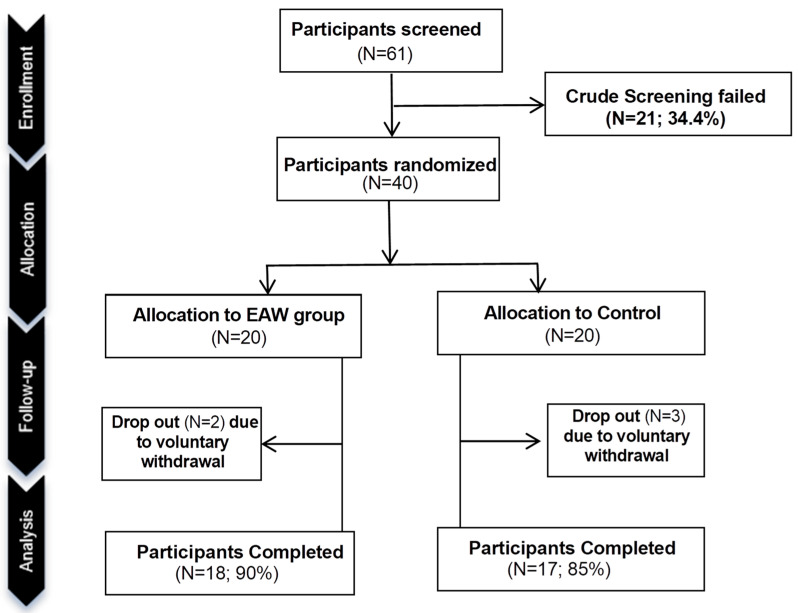
Consort diagram.

A total of 61 participants were initially screened for eligibility, of whom 21 (34.4%) did not meet the inclusion criteria during the preliminary screening (Figure 1). The remaining 40 eligible participants were subsequently randomized in a 1:1 ratio into the EAW intervention group (20 participants) and the control group (20 participants). During the intervention period, 2 participants (10%) in the EAW group and 3 participants (15%) in the control group withdrew for personal or non-study-related reasons. Ultimately, 18 participants (90%) in the EAW group and 17 participants (85%) in the control group completed the 12-week intervention and were included in the final analysis. The overall compliance rate achieved by the total study cohort was 91.90%. Notably, compliance was consistently high and did not differ significantly between groups (*p* = 0.6293). This finding confirms that the vast majority of participants strictly followed the study protocol, thereby reinforcing the reliability of our results.

Table S1 presents the baseline demographic and clinical characteristics of the study participants, stratified into two groups (Group A, 18 participants; Group B, 17 participants). The groups were well-balanced at baseline, with no statistically significant differences observed across all measured variables. Participants age (min–max: 18–59) was similar between EAW group (27 ± 9 years) and control group (26 ± 10 years) (*p* = 0.921), and the distribution of sex showed no significant difference (*p* = 0.338). All continuous clinical and laboratory parameters, including body mass index, diet purine intake, lipid profiles, liver function tests, renal function markers, uric acid levels, and glucose homeostasis indices, were comparable between the two groups, with all *p*-values exceeding 0.05. Similarly, the distributions of categorical variables from urinalysis, including white blood cells, red blood cells, casts, ketones, and glucose, showed no statistically significant differences between the groups (all *p* > 0.05).

The dietary results showed no significant changes in energy, fat, protein, carbohydrate, and purine intake within both the EAW group and the control group across the three time periods (Day 1–6, Day 7–12, Day 13–18), indicating that changes in other indicators originated from the intervention (Table S2).

### 3.2. Changes in Serum and Urinary Biomarkers

As shown in Table S1, at baseline, serum uric acid (SUA) levels were comparable between the alkaline water and control groups (467.20 ± 64.06 vs. 424.40 ± 64.18 μmol/L). During the 12-week intervention, SUA in the alkaline water group decreased progressively to 412.50 ± 77.71 μmol/L (Table 1). In contrast, the control group showed only a minor reduction (416.10 ± 79.12 μmol/L), with a significant between-group difference at week 12 (*p* = 0.04, linear mixed-effects model). Urinary uric acid (UUA) fluctuated within normal ranges without significant interaction effects (*p* > 0.05), suggesting that the urate-lowering effect was mainly reflected in serum levels. No significant between-group differences were observed in lipid profiles (TC, TG, HDL-C, LDL-C, FFA) or glucose–insulin parameters (FPG, FINS, HOMA-IR) throughout the intervention (*p* > 0.05), indicating that alkaline water did not affect lipid metabolism or glycemic homeostasis (Table S3). Among hepatic markers, AST levels showed transient between-group differences at week 4 (*p* = 0.014) and week 12 (*p* = 0.011), whereas ALT remained unchanged. Serum albumin (ALB) increased modestly in the alkaline water group but slightly declined in controls, with a significant difference at week 12 (*p* = 0.023). Importantly, despite this significant between-group difference in ALB change trends, ALB levels in both groups remained strictly within the clinical normal reference range over the entire study period.

Other hepatic indicators, including total protein, bilirubin fractions, ALP, and ALT/AST ratio, showed no significant alterations (*p* > 0.05). Renal function markers (BUN, Cr, eGFR) and urinary parameters (specific gravity, pH) remained stable and comparable between groups. A transient rise in total bile acid (TBA) at week 8 (*p* = 0.017) was not sustained by week 12.

Overall, 12 weeks of daily consumption of 1.5 L of electrolysed alkaline water significantly reduced serum uric acid levels and modestly increased serum albumin levels. Moreover, all monitored safety-related clinical parameters, including hepatic, or renal function, remained within normal reference ranges throughout the study period. No clinically relevant adverse events were observed in either group, indicating good metabolic and hepatic–renal safety of the intervention.

### 3.3. Changes in Health-Related Quality of Life (SF-36)

Scores of the Short-Form 36 Health Survey (SF-36) were used to evaluate changes in physical and mental health domains during the 12-week intervention. Overall, participants consuming alkaline water showed modest improvements in several physical domains compared with controls, whereas psychological dimensions remained essentially unchanged.

Specifically, Role-Physical (RP) and Bodily Pain (BP) scores increased slightly in the alkaline water group. They decreased in the control group, resulting in significant group-by-time interactions at week 12 (*p* = 0.008 and *p* = 0.041, respectively). The Physical Component Summary (PCS) also improved progressively in the alkaline water group (from 56.5 ± 5.2 to 59.9 ± 4.9). In contrast, a decline was observed in controls (from 56.6 ± 8.9 to 52.8 ± 9.4), yielding a significant difference at week 12 (*p* = 0.002). These findings indicate that consumption of alkaline water was associated with better perceived physical function and greater pain relief.

Other subscales—including Physical Functioning (PF), General Health (GH), Vitality (VT), Social Functioning (SF), Role-Emotional (RE), and Mental Health (MH)—showed no significant group-by-time interactions (*p* > 0.05), suggesting that the intervention did not markedly alter mental or social well-being within the 12 weeks. The Mental Component Summary (MCS) also remained stable across groups (*p* > 0.05).

Together, these results suggest that 12 weeks of daily intake of alkaline water significantly improved physical-related quality-of-life dimensions (RP, BP, and PCS), while maintaining mental health stability without adverse psychological effects.

### 3.4. Effect of EAW Treatment on the Gut Microbiota

To investigate the impact of EAW intervention on the gut microbial composition, fecal bacterial 16S rRNA gene sequencing was subsequently performed. The species rank–abundance and rarefaction curves indicated sufficient sequencing depth, high microbial richness, and a relatively even community distribution across all samples (Figure S1). As presented in Figures S2–S4, the α-diversity indices (Chao1, Observed features, Shannon, and Simpson) and β-diversity did not differ significantly before and after the intervention in either the EAW or control group, suggesting that the intervention exerted minimal influence on overall microbial diversity.

**Figure 2:**
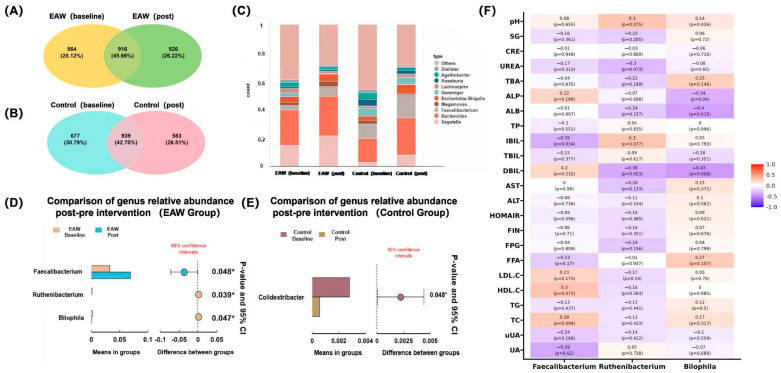
Gut microbial composition and differential analysis before and after intervention. (**A**,**B**) Venn diagrams showing the shared and unique genera between the baseline and post-intervention periods in the EAW and control groups; (**C**) Stacked bar plots of relative abundance at the genus level across groups; (**D**,**E**) Comparison of genus-level relative abundance before and after intervention in the EAW and control groups; (**F**) Heatmap showing correlations between key genera (*Faecalibacterium*, *Ruthenibacterium*, and *Bilophila*) and clinical parameters of the EAW group, the color bar represents the correlation coefficient: red corresponds to 1.0 (strong positive correlation), and blue corresponds to -1.0 (strong negative correlation). * indicates *p*-value < 0.05.

As shown in Figure 2, genus-level analyses revealed both shared and unique microbial features before and after intervention. In the EAW group, a total of 916 OTUs (45.66%) were shared between baseline and post-intervention samples, while 564 OTUs (28.12%) and 526 OTUs (26.22%) were unique to baseline and post-intervention, respectively (Figure 2A). In the control group, 939 OTUs (42.70%) were shared, with 677 (30.79%) and 583 (26.51%) unique to the baseline and post-intervention samples, respectively (Figure 2B).

To further elucidate the alterations in gut bacterial composition in response to EAW consumption, differential abundance analyses were performed to compare microbial profiles before and after the intervention. Among the top 15 genera with relative abundances greater than 1%, *Faecalibacterium* exhibited a significant increase after EAW intervention compared with baseline (*p* = 0.048). In contrast, no significant changes were observed in the control group. Other dominant genera, including *Bacteroides*, *Megamonas*, *Escherichia–Shigella*, *Roseburia*, and *Bifidobacterium* remained relatively stable across time points in both groups (*p* > 0.05) (Figure S5).

The genus-level composition remained generally similar across groups (Figure 2C); however, differential abundance analysis identified several genera that were significantly affected by the EAW intervention (Figure 2D, E). Specifically, the relative abundances of *Faecalibacterium* (*p* = 0.048, q = 0.674) and *Ruthenibacterium* (*p* = 0.039, q = 0.674) increased, whereas *Bilophila* (*p* = 0.047, q = 0.674) decreased after EAW consumption. In contrast, only *Colidextribacter* (*p* = −0.045, q = 0.524) showed a mild reduction in the control group. However, these changes were only observed before multiple-testing correction, but these did not remain significant thereafter (q > 0.05).

Correlation analysis (Figure 2F) demonstrated that these key genera exhibited distinct associations with clinical indicators, implying potential microbial contributions to the metabolic effects of EAW intervention. Specifically, the abundance of *Faecalibacterium* was significantly negatively correlated with serum uric acid (UA; r = −0.39, *p* = 0.02) and indirect bilirubin (IBIL; r = −0.35, *p* = 0.034), suggesting that its post-intervention increase may be associated with improved uric acid metabolism and reduced bilirubin levels. Additionally, *Faecalibacterium* showed borderline positive correlations with total cholesterol (TC; r = 0.28, *p* = 0.094) and high-density lipoprotein cholesterol (HDL-C; r = 0.3, *p* = 0.073), suggesting a potential, albeit modest, protective effect on lipid metabolism.

*Ruthenibacterium* showed a mild decrease following EAW intervention and was negatively correlated with direct bilirubin (DBIL; r = −0.38, *p* = 0.023). It also showed overall negative trends with lipid parameters, including LDL-C (r = −0.17, *p* = 0.33), HDL-C (r = −0.16, *p* = 0.354), TG (r = −0.13, *p* = 0.441), and TC (r = −0.13, *p* = 0.433), implying a possible association with improved lipid metabolism that requires further validation.

In contrast, *Bilophila* abundance significantly decreased after EAW intervention and was negatively correlated with alkaline phosphatase (ALP; r = −0.34, *p* = 0.04), albumin (ALB; r = −0.4, *p* = 0.015), and direct bilirubin (DBIL; r = −0.43 *p* = 0.008), suggesting that its reduction may be related to improved bile acid metabolism and enhanced hepatic protein synthesis.

### 3.5. Effect of EAW Treatment on the Gut Metabolic Profile

**Figure 3:**
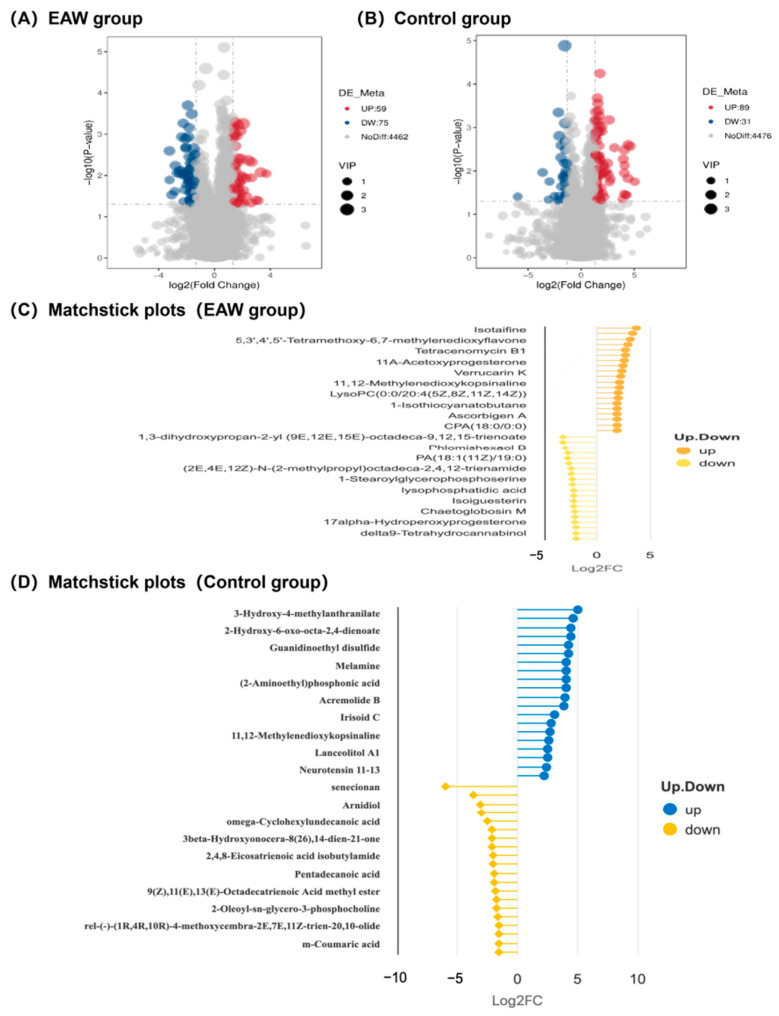
Differential gut metabolomic profiles and characteristic metabolites in the EAW and control groups before and after intervention (VIP > 1.0, fold change (FC) > 2.5 or <0.4, and *p* < 0.05) (**A**,**B**) Volcano plots showing significantly upregulated (red) and downregulated (blue) metabolites in the EAW and control groups; (**C**,**D**) Matchstick plots presenting the top differential metabolites ranked by logFC in the EAW and control groups.

As shown in Figure S6, PCA revealed substantial overlap between the EAW and control groups at baseline, indicating comparable metabolic profiles and well-balanced randomisation. PLS-DA analysis showed that in the control group, pre- and post-intervention samples partially overlapped with mild separation (R^2^ = 0.83, Q^2^ = −0.31), suggesting minimal metabolic drift. In contrast, the EAW group showed clear separation along PC1, with no overlap between ellipses (R^2^ = 0.85, Q^2^ = −0.37), indicating a distinct metabolic shift following EAW intervention (Figure S7). VIP scores derived from the PLS-DA model were further used to identify key differential metabolites. Based on the criteria of VIP > 1.0, fold change (FC) > 2.5 or <0.4, and *p* < 0.05, differential metabolites were identified between pre- and post-intervention samples (Figure 3). In the EAW group, 134 significant metabolites were detected among 4596 identified features, including 75 upregulated and 59 downregulated metabolites (Figure 3A, Table S4). Among these metabolites, 1,3-DIMETHYLURIC ACID, 2-Amino-5-chlorobenzoxazole, 4-guanidinobutanoate, 6-Acetamido-3-oxohexanoate, 9-Methyluric acid, Hecogenin, Methyl linolenate, Pristimerin, 8-O-Tigloyldiderroside, Flucytosine, Spicatolide E, and (2-Aminoethyl) phosphonic acid remained marginally significant (*p* < 0.1) following multiple-testing correction.

In contrast, the control group showed 120 significant metabolites, with 31 downregulated and 89 upregulated species (Figure 3B, Table S5). The matchstick plots (Figure 3C,D) highlight the top differential metabolites, ranked by logFC, and demonstrate distinct metabolic patterns between the two groups. The matchstick plots highlight the top differential metabolites, ranked by logFC, and demonstrate distinct metabolic patterns between the two groups. Among these metabolites, only Cidofovir remained marginally significant (*p* < 0.1) following multiple-testing correction.

**Figure 4:**
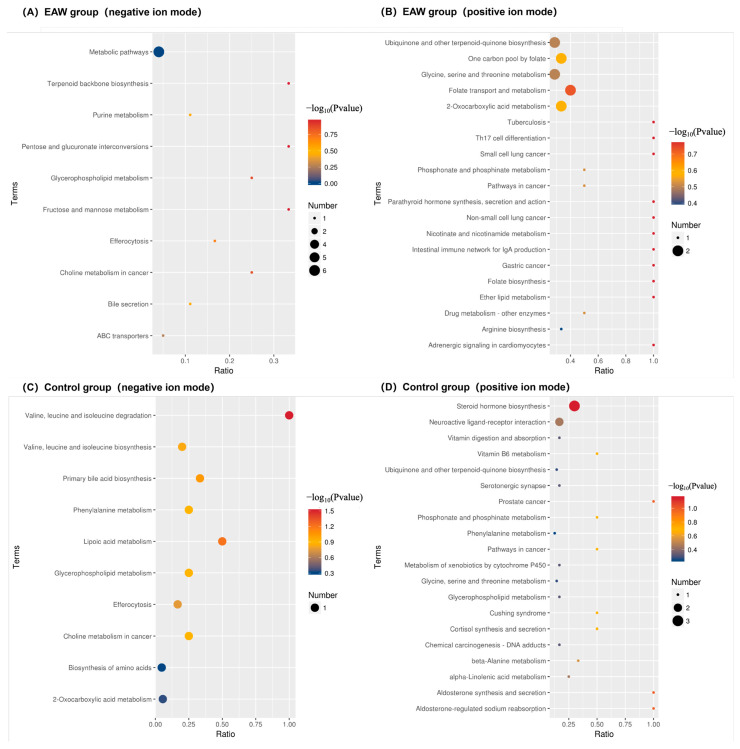
KEGG pathway enrichment analysis of differential metabolites before and after intervention. (**A**,**B**) Bubble plots showing enriched metabolic pathways of differential metabolites in the EAW group under positive- and negative-ion modes, respectively; (**C**,**D**) Bubble plots showing enriched metabolic pathways of differential metabolites in the control group under positive- and negative-ion modes, respectively. The bubble size represents the number of metabolites involved in each pathway, and the colour gradient indicates the significance level (−log_10_(*p*-value)), with larger, darker bubbles denoting more substantial enrichment.

To further explore the metabolic mechanisms underlying the uric acid–lowering effects of EAW, untargeted metabolomic data were analyzed under both positive- and negative-ion modes. In the negative-ion mode (Figure 4A), key enriched pathways included purine metabolism, the core route of uric acid synthesis, and fructose and mannose metabolism, suggesting that the intervention may reduce uric acid levels by regulating both purine turnover and upstream substrate supply. Pathways such as bile secretion, ABC transporters, and glycerophospholipid metabolism were also enriched, indicating that gut–liver interaction, transport mechanisms (e.g., ABCG2-mediated urate excretion), and improved metabolic homeostasis may collectively contribute to uric acid reduction.

In the positive-ion mode (Figure 4B), enrichment of 2-oxocarboxylic acid metabolism, folate metabolism, and glycine, serine and threonine metabolism suggested modulation of amino acid utilization and purine precursor availability.

In contrast, the control group showed markedly fewer enriched pathways and weaker associations with uric acid metabolism. In the negative-ion mode, enrichment was mainly observed in valine, leucine, and isoleucine degradation; lipoic acid metabolism; and primary bile acid biosynthesis, indicating mild alterations in amino acid and lipid metabolism without clear relevance to uric acid regulation (Figure 4C,D). In the positive-ion mode, the enriched pathways, such as steroid hormone biosynthesis, phosphate metabolism, and vitamin metabolism, reflected nonspecific metabolic fluctuations rather than targeted changes in purine- or fructose-related pathways.

## 4. Discussion

This randomized, double-blind, controlled clinical trial revealed that daily consumption of 1.5 L of EAW (pH 8.5–9.5) for 12 weeks significantly lowered serum uric acid (SUA) levels in adults with hyperuricemia, accompanied by improvements in physical health–related quality of life scores compared with the control group. Our findings are consistent with previous animal studies demonstrating that EAW effectively reduces uric acid and creatinine levels, enhances urinary urate excretion, and attenuates renal injury [18]. These observations suggest that EAW intake may be associated with alterations in gut metabolism, potentially related to reduced uric acid levels.

Previous clinical and experimental studies have demonstrated that an Alkali diet or the intake of alkaline water increases urinary pH and promotes uric acid excretion [27]. Additionally, EAW may influence the expression of urate transporters URAT1 and GLUT9 via its antioxidant and anti-inflammatory properties, which have been shown to improve renal function in murine models [18]. Collectively, these findings provide indirect support for a potential role of EAW in urate homeostasis. In addition, this study found that after 12 weeks of intervention, serum albumin levels increased slightly in the alkaline water group but decreased in the control group. These findings indicate that alkaline water intake had a significantly greater effect on maintaining or improving serum albumin levels than the control. Albumin is a key antioxidant protein and also a marker of nutritional and inflammatory status [28]. Studies have demonstrated that elevated serum uric acid levels can stimulate the release of inflammatory cytokines, such as interleukin-6 (IL-6) and tumour necrosis factor-α (TNF-α) [29], thereby inhibiting albumin synthesis. Elevated albumin concentrations may generally reflect preserved hepatic and renal function, lower systemic inflammation, and greater antioxidant capacity, all of which help maintain serum uric acid within a lower physiological range. Moreover, the concurrent decrease in serum uric acid and increase in albumin levels implies a reduction in the uric acid-to-albumin ratio, which allows for a more refined assessment of oxidative stress, inflammatory responses and nutritional status, and is a recently proposed biomarker of cardiovascular risk [30]. However, the changes in albumin and uric acid are mild, clinical implications of the observed reduction in this ratio of future cardiovascular risks require confirmation in larger and longer-term studies.

Emerging evidence suggests that the gut microbiota is involved in uric acid metabolism and intestinal urate handling, thereby contributing to systemic urate homeostasis [31]. Gut microbiota dysbiosis in hyperuricemia (HUA) is typically characterized by a decline in beneficial SCFA-producing taxa—such as *Faecalibacterium*, *Coprococcus*, and *Bifidobacterium*—and enrichment of pro-inflammatory or bile-tolerant genera including *Bilophila* and *Flavonifractor* [9,32]. In our study, we observed a modest trend toward increased *Faecalibacterium* and reduced *Bilophila* following EAW intake, though this pattern did not reach statistical significance after FDR adjustment. This finding aligns with multiple studies reporting a consistent depletion of *Faecalibacterium* in patients with hyperuricemia and gout [32,33]. *Faecalibacterium prausnitzii* is a primary butyrate-producing bacterium that exerts anti-inflammatory and immunomodulatory effects through butyrate-NF-κB signalling and tight-junction reinforcement, conferring protection against metabolic disorders such as obesity, diabetes, and hypertension. Therefore, the observed enrichment of *Faecalibacterium* may be linked to improved SCFA-mediated mucosal defence and reduced inflammatory tone, although causality cannot be inferred from the present data.

*Bilophila* is a bile-resistant, sulfite-reducing, anaerobic bacterium capable of utilizing taurine-conjugated bile acids as electron acceptors for energy metabolism. It proliferates preferentially under high-fat dietary conditions and has been consistently linked to intestinal inflammation and disruption of epithelial barrier integrity [32,34,35]. Thus, the reduction in *Bilophila* after EAW intake likely reflects improved gut barrier function and attenuation of mucosal inflammation. *Ruthenibacterium* has been less studied; however, recent studies have reported its enrichment in individuals with diseases and in the ageing population [36,37].

In the control group, *Colidextribacter* abundance decreased significantly after the intervention. *Colidextribacter* is known for its anti-inflammatory and lipid-modulating properties [38,39]. Several studies have demonstrated that higher *Colidextribacter* levels are inversely correlated with hepatic inflammation and serum total cholesterol. In contrast, a positive correlation has been observed between serum uric acid and renal function indices, including UA, XOD, BUN, CRE, MDA, URAT1, and GLUT9 [40]. Collectively, the concurrent increase in beneficial SCFA-producing taxa (e.g., *Faecalibacterium*) and decrease in pro-inflammatory or dysmetabolic genera (*Bilophila*, *Ruthenibacterium*) supports the hypothesis that EAW improves host metabolic health through microbiota-mediated alleviation of intestinal inflammation and restoration of gut–liver–kidney crosstalk.

In contrast, the control group showed limited microbial restructuring, and the decline in potentially protective taxa, Colidextribacter, may reflect the absence of beneficial modulation of metabolic and inflammatory pathways. Gut metabolomic analyses further corroborated the microbial findings, revealing that the differential metabolites were predominantly enriched in pathways related to purine metabolism, fructose and mannose metabolism, bile secretion, and ABC transporter systems—core processes intimately linked to urate generation and excretion. The coordinated modulation of these pathways suggests that EAW intervention not only reshaped the microbial composition but also altered gut microbial metabolism, which may reflect a gut environment more permissive to purine metabolism.

It should be noted that the microbiota and metabolomic analyses were exploratory in nature. Several associations did not remain statistically significant after false discovery rate (FDR) correction, and thus these findings should be interpreted as hypothesis-generating rather than confirmatory. Nonetheless, the observed microbial and metabolic patterns were biologically coherent and aligned with existing literature on hyperuricemia and gut dysbiosis.

The present study possesses several notable strengths. First, it is a randomized, double-blind, controlled human trial that systematically evaluated the urate-lowering efficacy of electrolysed alkaline water (EAW) in adults with hyperuricemia. Second, by integrating gut microbiota profiling with untargeted gut metabolomics, this study provides a comprehensive view of the gut ecological and metabolic responses to EAW intervention. The combined use of multi-omics analysis enables the identification of microbe–metabolite networks and metabolic pathways potentially involved in urate homeostasis. Third, the consistency between the observed microbial alterations and metabolic pathway shifts strengthens the biological plausibility of EAW-mediated improvements in urate regulation.

However, several limitations should be acknowledged. The sample size was relatively small, and the 12-week intervention period limits the generalization of long-term efficacy. Although strict randomization and blinding were employed to minimize bias, given the limited sample size, these findings should be interpreted as a pilot that warrants validation in larger-scale cohorts. Although this study revealed significant gut microbiota and metabolomic changes associated with urate reduction, causal relationships cannot be fully established based on observational omics data alone. Notably, while previous animal studies have demonstrated that EAW administration reduces serum uric acid and modulates renal urate transporters such as URAT1 and GLUT9, the causal mechanism linking EAW-induced gut microbial modulation to systemic urate metabolism remains to be experimentally verified. Future animal and mechanistic studies are warranted to determine whether the observed intestinal microbial shifts directly mediate urate metabolism and to further delineate the underlying biological mechanisms.

## 5. Conclusions

In summary, this 12-week pilot randomized controlled trial provides preliminary evidence that consumption of electrolyzed alkaline water was associated with modest reductions in serum uric acid and concurrent changes in gut microbial composition and metabolic profiles in adults with hyperuricemia. These multi-omics observations suggest that EAW may influence uric acid homeostasis through gut microbiota and its associated metabolic processes. Given its generally favourable tolerability and non-pharmacological nature, EAW could be considered a potential adjunctive approach for hyperuricemia management. Future mechanistic studies may integrate fecal microbiota transplantation and targeted pathway analyses to establish causality and inform subsequent clinical investigations.

## Figures and Tables

**Table 1:** Changes in uric acid level during 12 weeks of intervention in the alkaline water and control groups.

Variable	Group	Timepoint				*p* Value		
		Week 0	Week 4	Week 8	Week 12	Week 4	Week 8	Week 12
SUA (μmol/L)	EAW	467.20 ± 64.06	446.40 ± 67.12	440.60 ± 57.42	412.50 ± 77.71	0.17	0.176	0.04
	Control	424.40 ± 64.18	434.60 ± 75.99	428.30 ± 99.20	416.10 ± 79.12			
UUA (μmol/L)	EAW	3427.00 ± 1447.00	3644.00 ± 1585.00	4049.00 ± 1232.00	3623.00 ± 1558.00	0.874	0.123	0.563
	Control	3443.00 ± 1310.00	3756.00 ± 1586.00	3130.00 ± 1308.00	3990.00 ± 1398.00			

Note: Values are presented as mean ± SD unless otherwise specified. *p* values were derived from LMM analyses. Abbreviations: SUA, serum uric acid; UUA, urine uric acid.

## Data Availability

The data supporting the findings of this study are available from the corresponding author upon reasonable request.

## Supplementary Materials

The following supporting information can be downloaded at: https://www.mdpi.com/article/10.3390/nu18010107/s1; Table S1. Participants demographics and baseline characteristics; Table S2. Intake of Energy, Fat, Protein, Carbohydrate and Purine in EAW Group and Control Group at Different Time Periods; Table S3. Changes in clinical biochemical indices during 12 weeks of intervention in the alkaline water and control groups.; Table S4. Full list of identified metabolites with statistical analysis results of the EAW group; Table S5. Full list of identified metabolites with statistical analysis results of the control group; Figure S1. Rank–abundance curves (A) and rarefaction curves (B) of microbial communities across all samples; Figure S2. Comparison of α-diversity indices before and after intervention in the EAW group; Figure S3. Comparison of α-diversity indices before and after intervention in the control group; Figure S4. β-diversity analysis of the control group based on PCoA, showing community composition before (red, baseline) and after (blue, post-intervention) the intervention; Figure S5. Genus-level differences in the top 15 taxa (relative abundance > 1%) before and after intervention in the EAW and control groups; Figure S6. PCA and PLS-DA analyses of alkaline water intervention effects on gut health; Figure S7. Metabolic Pathway Enrichment Analysis of EAW Group and Control Group in Positive-Ion and Negative-Ion Modes.

## Abbreviations

The following abbreviations are used in this manuscript:

HUA	Hyperuricemia
SUA	Serum Uric Acid
EAW	Electrolyzed Alkaline Water
VAS	Visual Analogue Scale
SF-36	36-Item Short Form Survey
RCT	Randomized Controlled Trial
NSAIDs	Nonsteroidal Anti-Inflammatory Drugs
SCFA	Short-Chain Fatty Acid
BMI	Body Mass Index
ALT	Alanine Aminotransferase
AST	Aspartate Aminotransferase
eGFR	Estimated Glomerular Filtration Rate
CKD	Chronic Kidney Disease
TC	Total Cholesterol
TG	Triglycerides
HDL-C	High-Density Lipoprotein Cholesterol
LDL-C	Low-Density Lipoprotein Cholesterol
FFA	Free Fatty Acids
FPG	Fasting Plasma Glucose
FINS	Fasting Insulin
HOMA-IR	Homeostasis Model Assessment of Insulin Resistance
TP	Total Protein
ALB	Albumin
ALP	Alkaline Phosphatase
TBA	Total Bile Acid
BUN	Blood Urea Nitrogen
Cr	Creatinine
DBil	Direct Bilirubin
TBil	Total Bilirubin
IBil	Indirect Bilirubin
UUA	Urinary Uric Acid
SG	Specific Gravity
UpH	Urine pH
OTUs	Operational Taxonomic Units
LC-MS	Liquid Chromatography–Mass Spectrometry
UPLC-MS/MS	Ultra-Performance Liquid Chromatography-Tandem Mass Spectrometry
PCoA	Principal Coordinate Analysis
LEfSe	Linear Discriminant Analysis Effect Size
KEGG	Kyoto Encyclopedia of Genes and Genomes
PCA	Principal Component Analysis
PLS-DA	Partial Least Squares-Discriminant Analysis
VIP	Variable Importance in Projection
FC	Fold Change
MAR	Missing-At-Random
ML	Maximum Likelihood
URAT1	Urate Transporter 1
GLUT9	Glucose Transporter 9
XOD	Xanthine Oxidase
MDA	Malondialdehyde
ABC	ATP-Binding Cassette
ABCG2	ATP-Binding Cassette Superfamily G Member 2
