# Supplementary material for: A Pilot Randomized Controlled Trial and Multi-Omics Analysis of Electrolysed Alkaline Water: Impacts on Gut Microbiota and Metabolic Signatures in Hyperuricemia
Source: Nutrients. 2025 Dec 28;18(1):107. doi: 10.3390/nu18010107 (PMC12787813; doi:10.3390/nu18010107)
Supplement: Supplementary file 1 [file nutrients-18-00107-s001.zip › nutrients-4027817-supplementary.pdf]

Supplemental material

A Pilot Randomised Controlled Trial and Multi-Omics Analysis of Electrolysed Alkaline Water: Impacts on Gut Microbiota and Metabolic Signatures in Hyperuricemia

Tables:

Table S1. Participants demographics and baseline characteristics

Table S2. Intake of Energy, Fat, Protein, Carbohydrate and Purine in EAW Group and Control Group at Different Time Periods

Table S3. Changes in clinical biochemical indices during 12 weeks of intervention in the alkaline water and control groups.

Table S4. Full list of identified metabolites with statistical analysis results of the EAW group

Table S5. Full list of identified metabolites with statistical analysis results of the control group

Figures:

Figure S1. Rank–abundance curves (A) and rarefaction curves (B) of microbial communities across all samples

Figure S2. Comparison of α-diversity indices before and after intervention in the EAW group

Figure S3. Comparison of α-diversity indices before and after intervention in the control group

Figure S4. β-diversity analysis of the control group based on PCoA, showing community composition before (red, baseline) and after (blue, post-intervention) the intervention

Figure S5. Genus-level differences in the top 15 taxa (relative abundance >1%) before and after intervention in the EAW and control groups

Figure S6. PCA and PLS-DA analyses of alkaline water intervention effects on gut health

Figure S7. Metabolic Pathway Enrichment Analysis of EAW Group and Control Group in Positive-Ion and Negative-Ion Modes

Table S1. Participants demographics and baseline characteristics

Characteristic	group		p-value
	EAW group	Control group	
Age	27 ± 9	26 ± 10	0.921
Sex, n (%)			0.338
Male	17 (94.4%)	14 (82.4%)	
Female	1 (5.6%)	3 (17.6%)	
BMI	26.3 ± 3.1	25.2 ± 5.7	0.500
Purine (mg/day)	183 ± 204	131 ± 96	0.337
Serum_Uric_Acid	467 ± 64	424 ± 64	0.057
Urine_Uric_Acid	3,427 ± 1,447	3,443 ± 1,310	0.972
TC (mmol/L)	4.27 ± 1.02	4.52 ± 0.75	0.425
TG (mmol/L)	1.50 ± 0.95	1.37 ± 0.88	0.687
HDL-C (mmol/L)	1.08 ± 0.25	1.27 ± 0.37	0.085
LDL-C (mmol/L)	2.51 ± 0.70	2.57 ± 0.65	0.795
FFA (mmol/L)	0.48 ± 0.24	0.38 ± 0.16	0.167
FPG (mmol/L)	4.97 ± 0.52	5.60 ± 3.13	0.422
FINS (μIU/mL)	13 ± 14	12 ± 9	0.713
HOMA-IR	3.18 ± 3.98	3.95 ± 7.23	0.702
ALT (U/L)	33 ± 27	26 ± 18	0.326
AST (U/L)	22 ± 8	20 ± 8	0.709
ALT/AST	0.86 ± 0.33	0.97 ± 0.35	0.343
DBil (μmol/L)	1.99 ± 1.51	2.43 ± 1.71	0.425
TBil (μmol/L)	13.6 ± 6.6	14.9 ± 7.4	0.588
IBil (μmol/L)	11.6 ± 5.5	12.5 ± 5.9	0.658
TP (g/L)	72.4 ± 3.6	73.3 ± 5.7	0.580
ALB (g/L)	48.53 ± 2.56	49.76 ± 2.91	0.194
ALP (U/L)	85 ± 17	75 ± 28	0.190
TBA (μmol/L)	3.01 ± 1.28	4.16 ± 3.85	0.252
Urea	4.73 ± 1.03	4.75 ± 1.17	0.950
eGFR	123 ± 10	121 ± 14	0.615
Creatinine	69 ± 11	71 ± 15	0.785

Characteristic	group		p-value
	EAW group	Control group	
Specific_Gravity	1.019 ± 0.006	1.020 ± 0.006	0.673
Urine_Ph	6.36 ± 0.72	6.41 ± 0.71	0.836
WBC, n (%)			0.735
-	17 (94.4%)	15 (88.2%)	
+	1 (5.6%)	1 (5.9%)	
±	0 (0.0%)	1 (5.9%)	
Rbc, n (%)			0.486
-	16 (88.9%)	15 (88.2%)	
±	2 (11.1%)	0 (0.0%)	
++	0 (0.0%)	1 (5.9%)	
+++	0 (0.0%)	1 (5.9%)	
Uscme, n (%)			>0.999
-	15 (83.3%)	14 (82.4%)	
+	3 (16.7%)	3 (17.6%)	
Ketone, n (%)			0.486
-	18 (100.0%)	16 (94.1%)	
++	0 (0.0%)	1 (5.9%)	
Urine_Glucose, n (%)			0.486
-	18 (100.0%)	16 (94.1%)	
++	0 (0.0%)	1 (5.9%)	

Note: Values are presented as mean ± SD unless otherwise specified. P values were derived from Welch Two Sample t-test or Fisher's exact test

Abbreviations: TC, total cholesterol; TG, triglycerides; HDL-C, high-density lipoprotein cholesterol; LDL-C, low-density lipoprotein cholesterol; FFA, free fatty acids; FPG, fasting plasma glucose; FINS, fasting insulin; HOMA-IR, homeostasis model assessment of insulin resistance; ALT, alanine aminotransferase; AST, aspartate aminotransferase; ALT/AST, ALT-to-AST ratio; DBil, direct bilirubin; TBil, total bilirubin; IBil, indirect bilirubin; TP, total protein; ALB, albumin; ALP, alkaline phosphatase; TBA, total bile acids; BUN, blood urea nitrogen; Cr, creatinine; SG, specific gravity; UpH, urine pH; eGFR, estimated glomerular filtration rate.

Table S2. Intake of Energy, Fat, Protein, Carbohydrate and Purine in EAW Group and Control Group at Different Time Periods

	EAW group				Control group			
	Week 1-6	Week 7-12	Week 13-18	P	Week 1-6	Week 7-12	Week 13-18	P
Energy (kcal)	2 148 ± 418	2 095 ± 405	2 038 ± 392	0.080	2 102 ± 405	2 086 ± 410	2 071 ± 410	0.090
Fat (%)	32.1 ± 5.9	31.5 ± 5.7	30.9 ± 5.7	0.536	31.8 ± 6.0	31.4 ± 5.8	31.2 ± 5.8	0.642
Protein (%)	15.2 ± 2.8	14.8 ± 2.7	14.3 ± 2.6	0.456	15.0 ± 2.7	14.7 ± 2.6	14.5 ± 2.7	0.548
Carbonhydrate (%)	50.3 ± 7.2	51.0 ± 7.0	52.1 ± 7.0	0.394	50.8 ± 7.0	51.2 ± 7.1	51.6 ± 7.1	0.316
Purine (mg/day)	301 ± 88	299 ± 86	295 ± 85	0.717	298 ± 90	302 ± 89	303 ± 87	0.700
Fluid (ml)	1993.89±546.22	2051.46±632.57	1985.85 ±625.82	0.582	1939.80±515.46	1890.6±515.03	1885.96±539.02	0.640

Table S3. Changes in clinical biochemical indices during 12 weeks of intervention in the alkaline water and control groups.

Variable	EAW group				Control group				P value		
	week0	week4	week8	week12	week0	week4	week8	week12			
TC (mmol/L)	4.27 ± 1.02	4.41 ± 1.09	4.23 ± 0.97	4.46 ± 1.17	4.52 ± 0.75	4.55 ± 0.67	4.37 ± 0.70	4.52 ± 0.56	0.513	0.514	0.245
TG (mmol/L)	1.50 ± 0.96	1.45 ± 0.93	1.52 ± 1.00	1.67 ± 1.29	1.37 ± 0.88	1.38 ± 1.13	1.64 ± 1.66	1.42 ± 1.28	0.788	0.262	0.586
HDL-C (mmol/L)	1.08 ± 0.25	1.10 ± 0.22	1.03 ± 0.26	1.07 ± 0.26	1.27 ± 0.37	1.32 ± 0.41	1.20 ± 0.36	1.21 ± 0.37	0.528	0.687	0.348
LDL-C (mmol/L)	2.51 ± 0.70	2.55 ± 0.72	2.44 ± 0.64	2.78 ± 0.86	2.57 ± 0.65	2.52 ± 0.65	2.45 ± 0.64	2.74 ± 0.57	0.454	0.647	0.397
FFA (mmol/L)	0.48 ± 0.24	0.53 ± 0.24	0.45 ± 0.27	0.49 ± 0.34	0.38 ± 0.16	0.47 ± 0.22	0.44 ± 0.23	0.49 ± 0.23	0.576	0.234	0.184
FPG (mmol/L)	4.97 ± 0.52	5.22 ± 0.48	5.29 ± 0.82	5.25 ± 0.52	5.60 ± 3.13	5.46 ± 1.90	5.82 ± 2.78	5.79 ± 2.30	0.196	0.726	0.737
FINS (μIU/mL)	13.48 ± 13.86	10.38 ± 9.48	14.84 ± 16.56	18.61 ± 24.55	12.01 ± 9.23	8.93 ± 5.26	18.03 ± 31.49	18.35 ± 23.90	0.998	0.504	0.863
HOMA-IR	3.18 ± 3.98	2.53 ± 2.65	3.83 ± 5.21	4.45 ± 5.95	3.95 ± 7.23	2.41 ± 2.51	5.94 ± 13.49	5.98 ± 10.01	0.734	0.61	0.773
ALT (U/L)	33.33 ± 26.94	33.47 ± 26.18	34.94 ± 25.63	31.61 ± 21.14	25.59 ± 18.33	20.65 ± 13.54	18.94 ± 8.90	18.88 ± 9.39	0.196	0.036	0.205
AST (U/L)	21.50 ± 7.63	23.39 ±	22.56 ± 7.43	23.83 ± 8.60	20.47 ± 8.48	17.59 ± 6.37	18.06 ± 3.85	17.88 ± 3.50	0.014	0.073	0.01

Variable	EAW group				Control group				P value		
	week0	week4	week8	week12	week0	week4	week8	week12			
		10.78									1
ALT/AST	0.86 ± 0.33	0.89 ± 0.33	0.87 ± 0.41	0.94 ± 0.42	0.97 ± 0.35	0.99 ± 0.33	1.10 ± 0.38	1.12 ± 0.40	0.91	0.174	0.419
DBil (μmol/L)	1.99 ± 1.52	2.72 ± 1.79	3.36 ± 4.63	5.44 ± 2.94	2.44 ± 1.71	2.55 ± 1.76	3.58 ± 2.06	6.66 ± 2.56	0.481	0.797	0.368
TBil (μmol/L)	13.59 ± 6.64	13.57 ± 4.95	12.25 ± 7.05	10.88 ± 5.43	14.89 ± 7.40	13.24 ± 4.74	12.68 ± 3.88	14.19 ± 5.52	0.351	0.62	0.252
IBil (μmol/L)	11.59 ± 5.46	10.87 ± 3.50	8.91 ± 4.07	5.44 ± 2.65	12.45 ± 5.89	10.69 ± 3.20	9.12 ± 3.63	7.54 ± 3.00	0.458	0.645	0.376
TP (g/L)	72.42 ± 3.56	74.28 ± 4.56	70.91 ± 3.36	70.31 ± 3.80	73.33 ± 5.72	74.59 ± 3.89	72.34 ± 3.56	69.62 ± 3.54	0.583	0.632	0.144
ALB (g/L)	48.53 ± 2.56	50.84 ± 2.96	48.39 ± 2.03	49.47 ± 2.73	49.76 ± 2.91	51.33 ± 1.55	48.68 ± 2.67	49.03 ± 1.89	0.314	0.201	0.023
ALP (U/L)	85.06 ± 16.82	90.03 ± 18.78	91.33 ± 22.49	88.39 ± 20.36	74.53 ± 27.84	79.47 ± 19.38	80.82 ± 19.07	76.24 ± 17.94	0.994	0.997	0.683
TBA (μmol/L)	3.01 ± 1.28	3.04 ± 1.57	5.26 ± 4.04	4.46 ± 3.00	4.17 ± 3.85	4.27 ± 2.44	3.98 ± 1.90	4.98 ± 3.32	0.943	0.017	0.531
BUN (mmol/L)	4.73 ± 1.03	5.16 ± 1.14	4.78 ± 0.88	5.14 ± 1.29	4.76 ± 1.17	5.34 ± 1.16	4.40 ± 1.10	4.74 ± 1.00	0.674	0.3	0.266
Cr (μmol/L)	69.39 ± 10.79	66.83 ± 11.36	66.17 ± 9.87	70.83 ± 10.21	70.59 ± 14.63	68.88 ± 15.61	67.47 ± 15.32	72.18 ± 16.09	0.672	0.867	0.822

Variable	EAW group				Control group				P value		
	week0	week4	week8	week12	week0	week4	week8	week12			
SG ((no unit))	1.02 ± 0.01	1.02 ± 0.01	1.02 ± 0.01	1.02 ± 0.01	1.02 ± 0.01	1.02 ± 0.00	1.02 ± 0.01	1.02 ± 0.00	0.794	0.974	0.965
UpH ((no unit))	6.36 ± 0.72	6.25 ± 0.52	6.39 ± 0.72	6.17 ± 0.54	6.41 ± 0.71	6.18 ± 0.59	6.03 ± 0.45	6.21 ± 0.50	0.925	0.941	0.55
eGFR (mL/min/1.73 m2)	123.00 ± 10.24	125.00 ± 11.18	125.80 ± 6.93	122.10 ± 11.55	120.90 ± 13.64	121.50 ± 17.27	124.30 ± 13.02	120.80 ± 14.46	0.622	0.104	0.964

Note: Values are presented as mean ± SD unless otherwise specified. P values were derived from LMM analyses.

Abbreviations: TC, total cholesterol; TG, triglycerides; HDL-C, high-density lipoprotein cholesterol; LDL-C, low-density lipoprotein cholesterol; FFA, free fatty acids; FPG, fasting plasma glucose; FINS, fasting insulin; HOMA-IR, homeostasis model assessment of insulin resistance; ALT, alanine aminotransferase; AST, aspartate aminotransferase; ALT/AST, ALT-to-AST ratio; DBil, direct bilirubin; TBil, total bilirubin; IBil, indirect bilirubin; TP, total protein; ALB, albumin; ALP, alkaline phosphatase; TBA, total bile acids; BUN, blood urea nitrogen; Cr, creatinine; SG, specific gravity; UpH, urine pH; eGFR, estimated glomerular filtration rate.

Figure S1. Rank–abundance curves (A) and rarefaction curves (B) of microbial communities across all samples

(A)

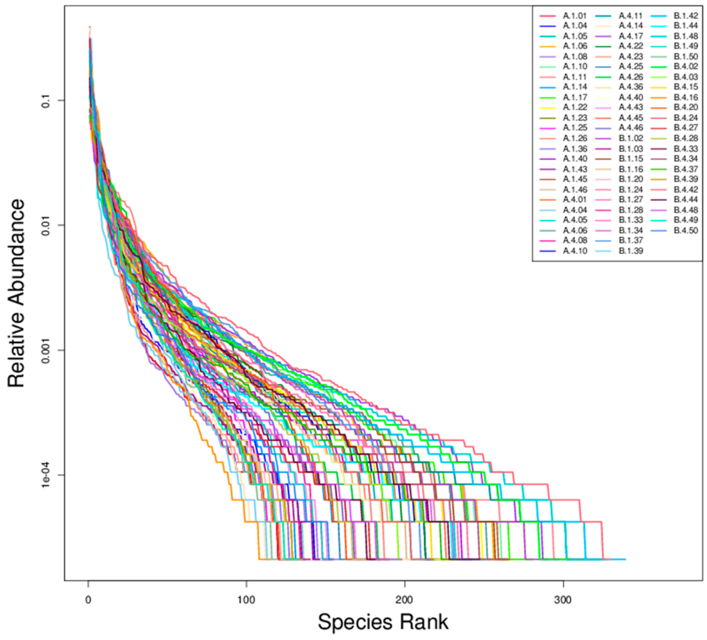

(B)

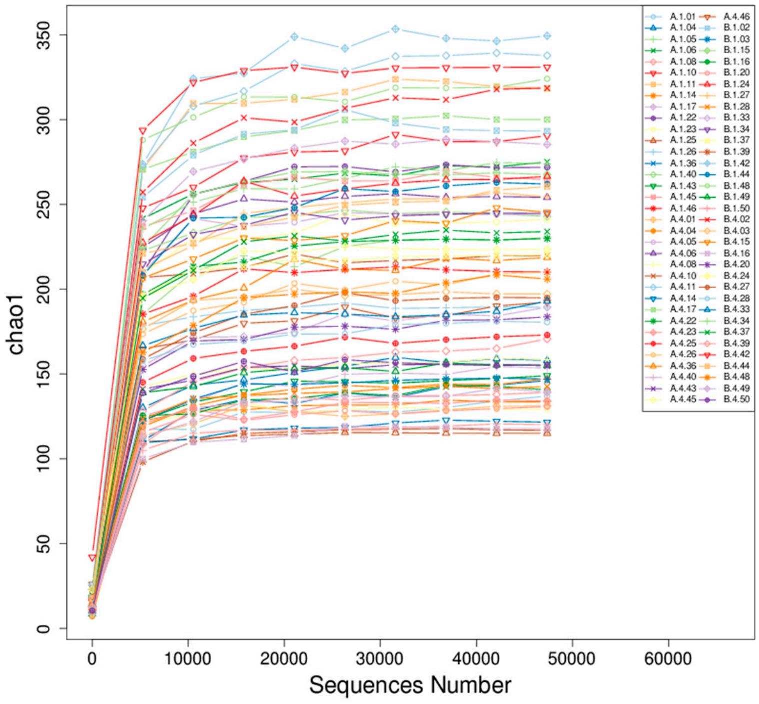

Figure S2. Comparison of α-diversity indices before and after intervention in the EAW group

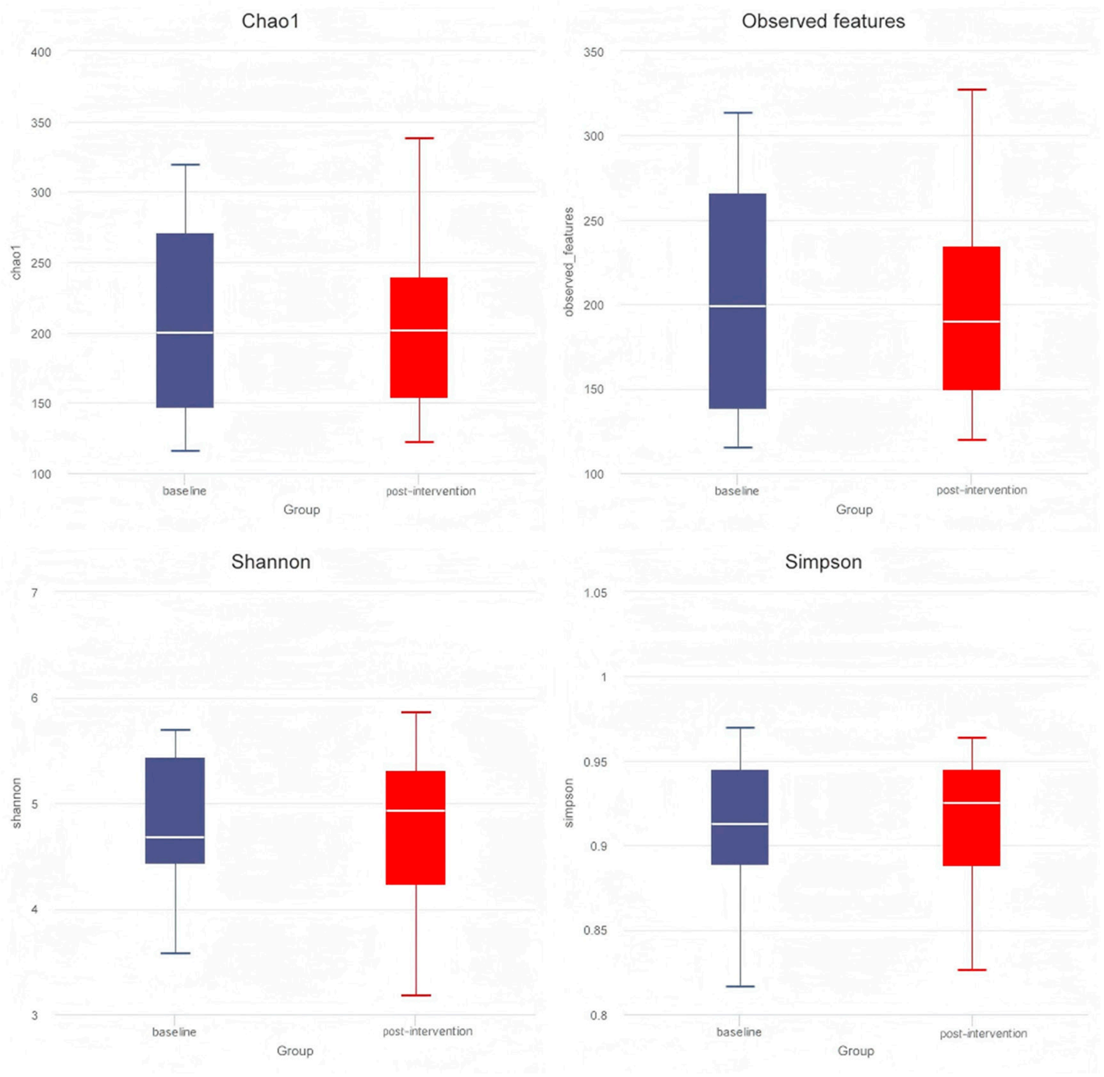

Figure S3. Comparison of α-diversity indices before and after intervention in the control group

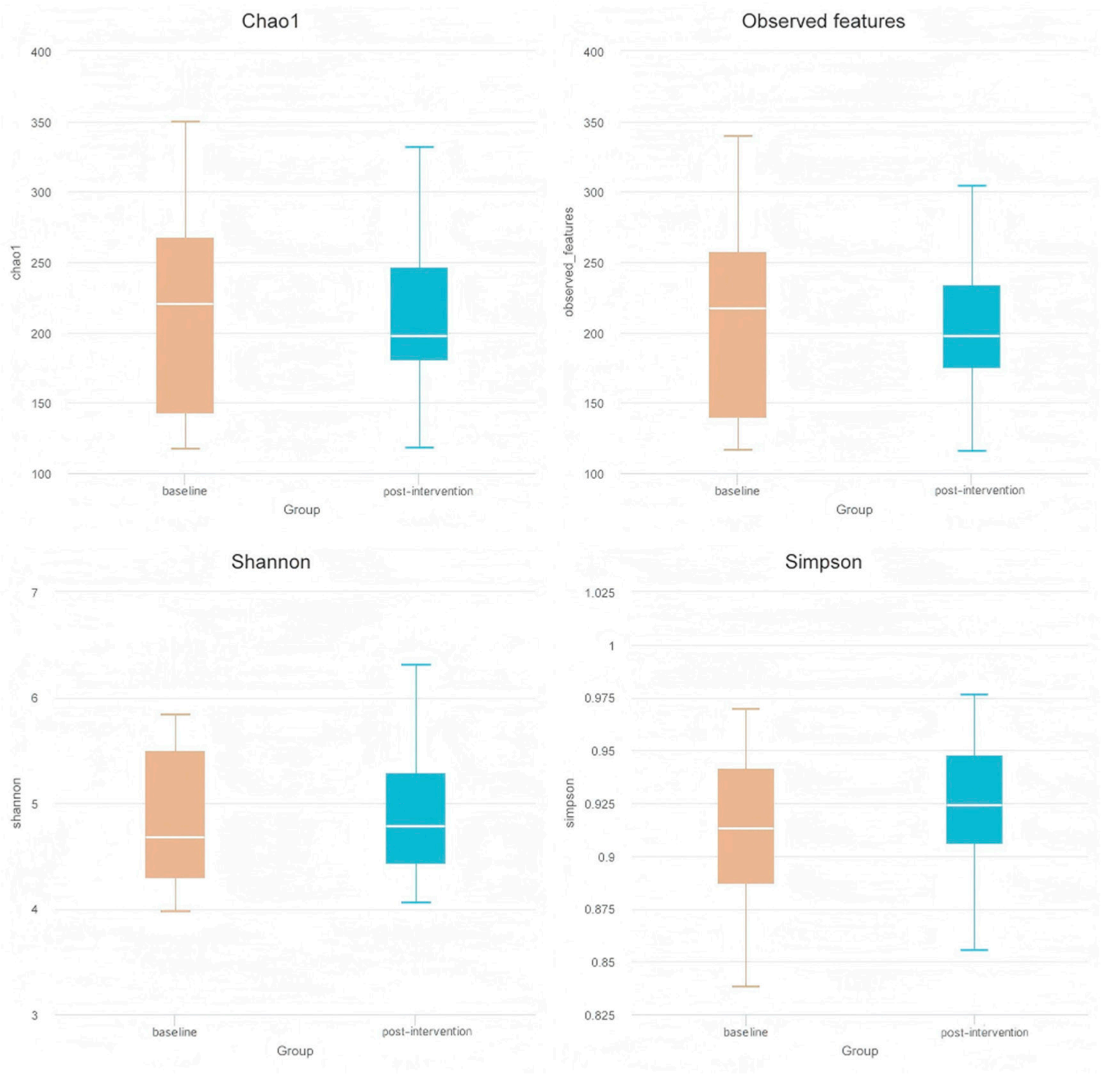

Figure S4. β-diversity analysis of the control group based on PCoA, showing community composition before (red, baseline) and after (blue, post-intervention) the intervention

（A）**EAW group**

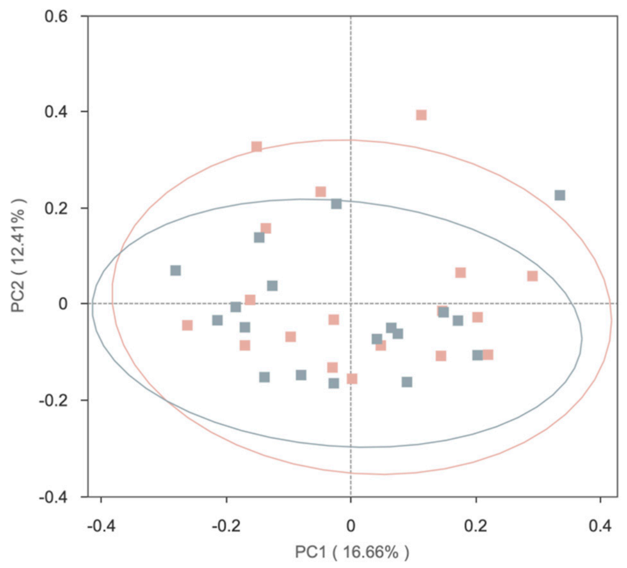

（B）**Control Group**

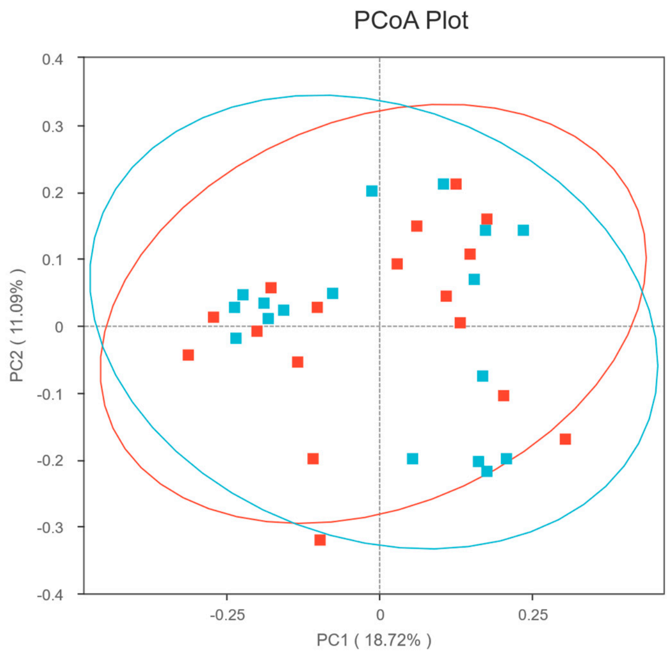

Figure S5. Genus-level differences in the top 15 taxa (relative abundance >1%) before and after intervention in the EAW and control groups.

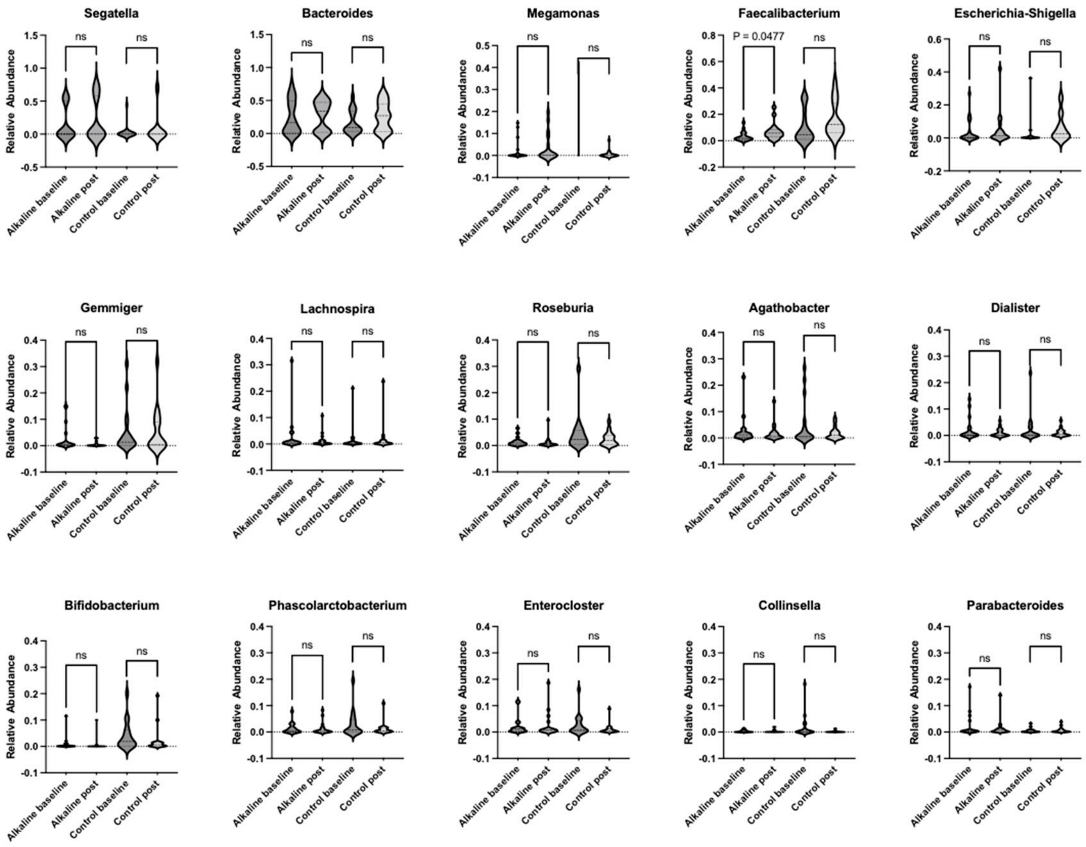

Figure S6. Principal component analysis (PCA) plots showing gut metabolic profile differences between the EAW and control groups at baseline

(A) .Negative ion mode

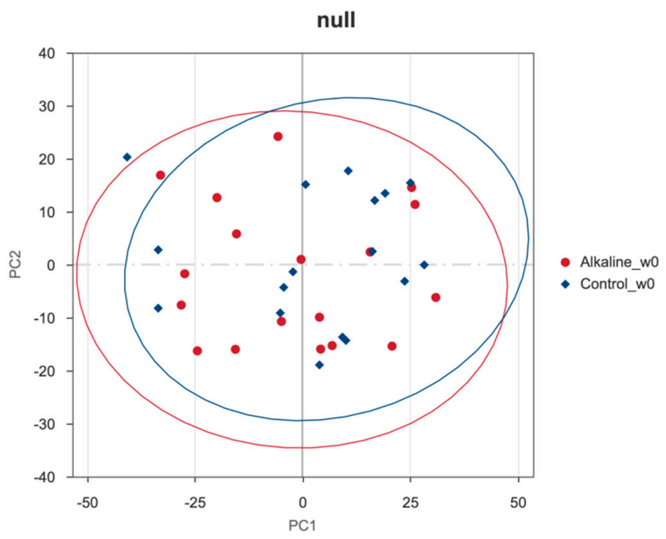

(B) Positive ion mode

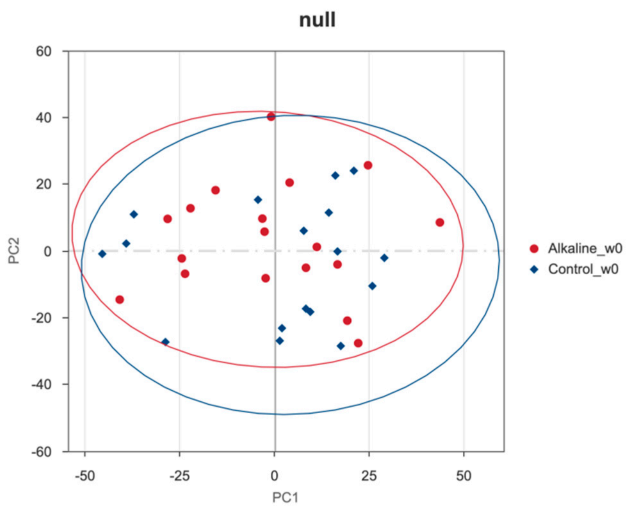

Figure S7. PCA and PLS-DA analyses of alkaline water intervention effects on gut health

(A) EAW group

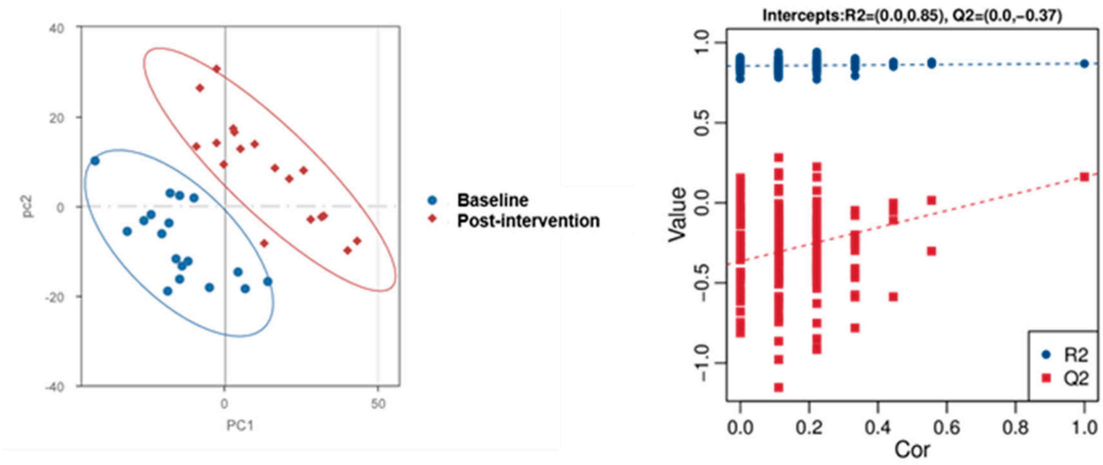

(B) Control group

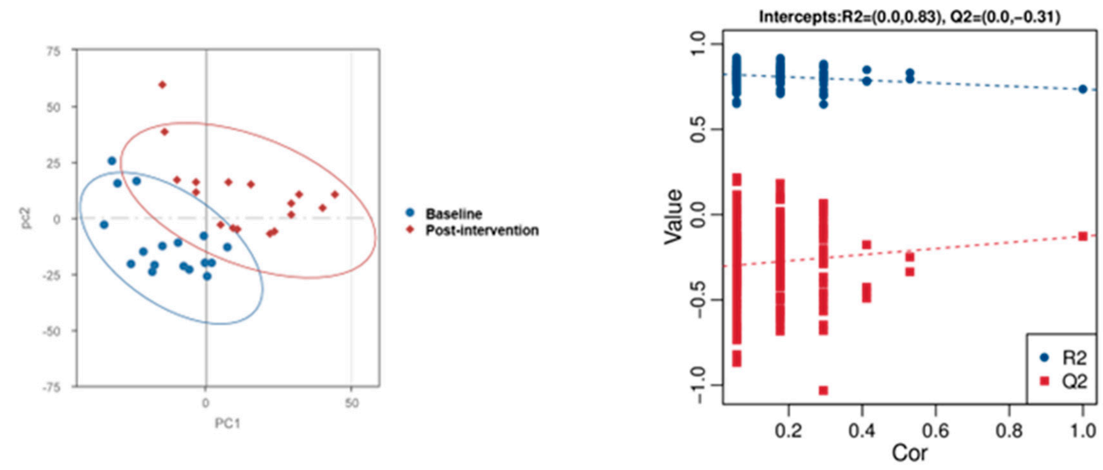

Table S4. Full list of identified metabolites with statistical analysis results of the EAW group

Metabolites	FC	Direct	pvalu	VIP	qvalu
1,3-DIMETHYLURIC ACID	4.161	up	0.000	3.04	0.086
2-Amino-5-chlorobenzoxazole	2.633	up	0.000	1.56	0.086
4-guanidinobutanoate	3.239	up	0.000	1.99	0.086
6-Acetamido-3-oxohexanoate	2.772	up	0.000	1.93	0.086
9-Methyluric acid	2.500	up	0.000	1.79	0.086
Hecogenin	0.284	down	0.000	1.61	0.086
Methyl linolenate	0.322	down	0.000	1.84	0.086
Pristimerin	0.212	down	0.000	1.55	0.086
8-O-Tigloyldiderroside	3.635	up	0.000	1.81	0.088
Flucytosine	2.770	up	0.000	1.84	0.095
Spicatolide E	0.266	down	0.000	2.77	0.097
(2-Aminoethyl)phosphonic acid	2.571	up	0.001	1.58	0.098
p-Chlorobenzenesulfonamide	3.005	up	0.001	1.68	0.100
1,3,7-TRIMETHYLURIC ACID	2.547	up	0.001	1.78	0.101
2,6-Dimethyl-3,6-octadiene-1,2,8-triol	2.847	up	0.001	1.63	0.119
delta9-Tetrahydrocannabinol	0.242	down	0.002	1.45	0.119
1,3-dihydroxypropan-2-yl	0.107	down	0.002	1.74	0.135
alpha-Tocospiro B	0.379	down	0.003	1.26	0.142
6-cis-Docosenamide	0.369	down	0.004	1.44	0.148
Apo-10'-zeaxanthinal	0.280	down	0.003	1.43	0.148
Ascorbigen A	3.736	up	0.003	2.00	0.148
N-METHYLCYCLOHEXYLAMINE	3.180	up	0.003	1.96	0.148
Tetracenomycin B1	6.367	up	0.004	1.80	0.152
2,4,8-Eicosatrienoic acid isobutylamide	0.313	down	0.004	2.36	0.154
Fumarylcarboxamido-L-2,3-diaminopropionyl-L-	3.140	up	0.004	1.50	0.154
N-acetyl putrescine	2.775	up	0.005	1.47	0.154
2-hydroxyoctanedioic Acid	2.508	up	0.007	1.49	0.156
Aspochalasin C	0.361	down	0.007	1.33	0.156
Corymbi-7,13E-dienolide	0.174	down	0.007	1.37	0.156
Decaethylene glycol	2.839	up	0.005	1.33	0.156
delta-Tocotrienol	0.306	down	0.006	1.30	0.156
Lyso-PAF C-18	0.285	down	0.007	1.24	0.156
N-Isobutyl-(2E,4E)-octadecadienamide	0.305	down	0.006	1.63	0.156
Robustamine cis-N-oxide	2.941	up	0.007	1.21	0.156
Brucine	10.00	up	0.008	2.13	0.159
(2E,4E,12Z)-N-(2-methylpropyl)octadeca-2,4,12-	0.174	down	0.009	1.34	0.164
Isotaifine	13.12	up	0.009	1.19	0.164
1-Stearoyl-sn-glycero-3-phosphocholine	0.251	down	0.009	1.18	0.165

Metabolites	FC	Direct	pvalu	VIP	qvalu
1-Isothiocyanatobutane	3.773	up	0.010	1.57	0.169
Monoolein	0.268	down	0.010	1.36	0.169
LysoPC(0:0/20:4(5Z,8Z,11Z,14Z))	4.202	up	0.010	1.42	0.172
N-(1-Deoxy-1-fructosyl)leucine	6.315	up	0.011	1.45	0.174
13,17-Epoxy-16-hydroxy macrolactin A	4.806	up	0.011	1.42	0.178
Ulipristal acetate	0.107	down	0.012	1.26	0.185
Protoporphyrin IX	0.337	down	0.013	1.32	0.187
Methyl palmitate	0.364	down	0.013	1.19	0.187
N-Lauroyl Methionine	0.300	down	0.013	1.54	0.187
PHOSPHOCHOLINE	0.325	down	0.013	1.09	0.187
sn-Glycero-3-phosphocholine	3.584	up	0.014	1.33	0.187
D-1-[(3-Carboxypropyl)amino]-1-deoxyfructose	3.484	up	0.015	1.31	0.193
10-Oxabenzo[def]chrysen-9-one	3.088	up	0.015	1.23	0.194
N-Oleoyl Arginine	0.353	down	0.016	1.05	0.199
3b-Hydroxy-5-cholenoic acid	0.251	down	0.016	1.33	0.200
Valyllysine	2.941	up	0.016	1.24	0.200
Ethiofencarb	0.380	down	0.017	1.59	0.201
cis-13,16-Docosadienoic acid	0.321	down	0.019	1.70	0.210
Cordycepin	2.630	up	0.019	1.21	0.210
N-Acetylornithine	2.718	up	0.022	1.30	0.215
N-Docosahexaenoyl Leucine	3.162	up	0.022	1.31	0.215
Phenylalanine deoxycholic acid	2.592	up	0.021	1.36	0.215
1-(2,3-Dihydro-6,7-dimethyl-1H-pyrrolizin-5-yl)-	4.242	up	0.023	1.43	0.215
MG(0:0/18:1(9Z)/0:0)	0.327	down	0.024	1.12	0.216
Ganoderic acid D	0.298	down	0.024	1.02	0.219
S-Allyl-L-cysteine	3.675	up	0.027	1.45	0.226
Phlomishexaol B	0.119	down	0.029	1.41	0.229
2-Chlorooctadecanoic acid	2.974	up	0.029	1.20	0.230
Verrucarin K	4.965	up	0.031	1.10	0.237
MG(0:0/21:0/0:0)	0.372	down	0.033	1.23	0.243
5-Pentadecylresorcinol	0.389	down	0.033	1.16	0.244
2-Amino-4-hydroxy-6,7-dimethyl-5,6,7,8-tetrahy	2.903	up	0.036	1.11	0.251
CPA(18:0/0:0)	3.649	up	0.035	1.13	0.251
gamma-Tocotrienol	0.296	down	0.035	1.20	0.251
3beta,4alpha,13alpha-Trihydroxylupanine	3.321	up	0.037	1.44	0.252
Holstiline	7.797	up	0.039	1.37	0.257
Tetradecanoylcarnitine	0.373	down	0.040	1.14	0.257
Octadecyl fumarate	0.294	down	0.040	1.13	0.260
7-Methoxydeoxymorellin	2.701	up	0.042	1.17	0.269
17alpha-Hydroperoxyprogesterone	0.236	down	0.001	2.61	0.269
Avocadyne Acetate	0.197	down	0.001	2.39	0.269
Kermadecin G	0.269	down	0.001	2.98	0.269

Metabolites	FC	Direct	pvalu	VIP	qvalu
(24Z)-29-Hydroxy-24(28)-dehydromakisterone C	3.352	up	0.045	1.06	0.275
11A-Acetoxyprogesterone	6.190	up	0.046	1.53	0.275
Hyperidixanthone	3.109	up	0.046	1.09	0.276
Wilforlide A	0.351	down	0.047	1.40	0.279
Isovouacapenol E	3.051	up	0.048	1.29	0.279
1-Stearoyl-sn-glycero-3-phosphate	0.241	down	0.002	2.22	0.361
alpha-Kudinlactone	0.353	down	0.002	2.66	0.361
Butanoic acid, [(diethoxyphosphinyl)oxy]methyl	0.349	down	0.003	2.08	0.466
Lauryl sulfate	5.349	up	0.004	2.71	0.493
2-Hydroxydocosanoic acid	0.320	down	0.005	2.27	0.510
PA(18:1(11Z)/19:0)	0.143	down	0.005	2.31	0.510
1-Stearoylglycerophosphoserine	0.194	down	0.007	1.98	0.548
Isotingenone III	0.155	down	0.008	2.09	0.567
lysophosphatidic acid	0.206	down	0.008	1.74	0.567
2-(6-oxo-1-oxaspiro[2.5]octa-4-glucosyl)-6-hydro	0.398	down	0.013	2.20	0.576
2-Hydroxyphytanic acid	0.276	down	0.010	2.32	0.576
Chaetoglobosin M	0.224	down	0.010	1.89	0.576
Epoxycladine C	0.388	down	0.013	1.97	0.576
Griffonianone C	0.280	down	0.012	1.78	0.576
Isoiguesterin	0.217	down	0.009	2.06	0.576
N-Docosahexaenoyl GABA	0.290	down	0.013	1.85	0.576
Palmitoylglycine	0.348	down	0.012	2.05	0.576
Tungtungmadic acid	2.875	up	0.010	2.54	0.576
19,20-DiHDPA	0.277	down	0.014	1.71	0.589
3-Hydroxycinnamoylglycine sulfate	2.927	up	0.014	1.89	0.594
2-Hydroxyhexadecanoic acid	0.370	down	0.017	2.24	0.620
CPA(18:1(11Z)/0:0)	0.392	down	0.016	1.70	0.620
LysoPS(16:0/0:0)	0.344	down	0.017	1.78	0.620
Kushenol G	0.361	down	0.020	2.00	0.631
LPC 18:3	0.139	down	0.024	1.57	0.631
moclobemide	0.310	down	0.025	2.00	0.631
Muscol	0.374	down	0.023	2.08	0.631
N-Methylcorydaldine	2.656	up	0.025	1.48	0.631
PGP(17:0cycw7/15:0cycw5)	3.261	up	0.021	2.20	0.631
Schweinfurthin B	2.571	up	0.022	1.75	0.631
Trimetaphosphoric acid	0.230	down	0.021	2.09	0.631
Uric acid	0.397	down	0.021	1.78	0.631
DIBOA	3.008	up	0.028	1.75	0.640
N-Acetyl-4-O-acetylneuraminic acid	3.068	up	0.031	1.76	0.653
9-F1-phytoprostane	0.394	down	0.034	1.61	0.696
Isoastragaloside I	2.915	up	0.038	2.00	0.718
(+)-O-Methyldihydrobotrydial	3.757	up	0.047	1.84	0.724

Metabolites	FC	Direct	pvalu	VIP	qvalu
5,3',4',5'-Tetramethoxy-6,7-methylenedioxyflavon	8.891	up	0.040	1.89	0.724
ent-kaur-16-ene	0.295	down	0.039	1.85	0.724
Formebolone	0.352	down	0.047	1.83	0.724
Hexanal octane-1,3-diol acetal	0.303	down	0.040	2.00	0.724
Hydroxymethoxyphenylcarboxylic	2.640	up	0.040	1.46	0.724
Oxymetholone	0.359	down	0.047	1.46	0.724
Stearoyllactic acid	0.338	down	0.042	1.56	0.724
Tetrahydropersin	0.217	down	0.043	1.90	0.724
PE(16:0/18:2(9Z,12Z))	0.310	down	0.048	1.68	0.728
MG(0:0/18:1(11Z)/0:0)	0.335	down	0.035	1.15	0.251
11,12-Methylenedioxykopsinaline	4.465	up	0.046	1.07	0.276
2-Oleoyl-sn-glycero-3-phosphocholine	0.298	down	0.047	1.02	0.279

Table S5. Full list of identified metabolites with statistical analysis results of the control group

Metabolites	FC	Direct	pvalu	VIP	qvalu
Cidofovir	0.343	down	0.000	4.166	0.025
Ile Leu Leu	3.416	up	0.000	2.062	0.151
Ile Phe Leu	2.885	up	0.000	2.024	0.168
Breyniaionoside C	2.989	up	0.000	1.945	0.168
Ile Ile Leu	3.123	up	0.000	2.634	0.255
Phe Ala Leu	2.623	up	0.000	2.198	0.168
3beta-Hydroxyonocera-8(26),14-dien-21-one	0.227	down	0.000	2.183	0.168
Leu Lys Phe	3.286	up	0.000	1.760	0.168
Lidamidine	6.235	up	0.000	2.044	0.168
Epothilone B10	2.510	up	0.000	1.614	0.168
Lys Ile Leu	3.003	up	0.000	1.945	0.168
Vaccenic acid	0.349	down	0.000	3.057	0.255
Sedumoside I	3.154	up	0.000	2.036	0.168
Phe Phe Leu	3.727	up	0.000	2.328	0.168
Val Leu Phe	3.015	up	0.001	1.800	0.168
Knightalbinol	3.791	up	0.001	2.177	0.168
Val Ile Leu	3.241	up	0.001	1.920	0.168
Ala-Glu-OH	2.600	up	0.001	2.101	0.168
Platanionoside I	2.755	up	0.001	1.851	0.168
Ala Ile Ile	2.573	up	0.001	1.714	0.168
Ile Phe Thr	2.583	up	0.001	1.759	0.168
Docosanamide	0.238	down	0.001	2.660	0.168
Lys Val Phe	2.557	up	0.001	1.678	0.168
4,4-Difluoropregn-5-ene-3,20-dione	2.722	up	0.001	1.900	0.191
m-Coumaric acid	0.348	down	0.002	2.584	0.368
2-Hydroxy-6-oxo-octa-2,4-dienoate	21.51	up	0.002	2.050	0.191
Pentadecanoic acid	0.265	down	0.002	2.511	0.417
Leu Ala Lys	3.201	up	0.002	1.740	0.193
Xanthopterin	24.46	up	0.002	1.960	0.193
Irisoid C	8.729	up	0.002	2.460	0.427
Roburic acid	0.353	down	0.003	1.899	0.195
(2-Aminoethyl)phosphonic acid	16.65	up	0.003	1.998	0.195
N-Eicosapentaenoyl Leucine	2.602	up	0.004	1.920	0.211
Ala-Leu-Leu-Asp	2.590	up	0.004	1.565	0.211
Dehydrolycopecurine	3.286	up	0.004	1.567	0.213
Asn Val Leu	3.019	up	0.004	1.609	0.213
Emopamil	0.266	down	0.004	1.990	0.216
Acremolide B	15.59	up	0.005	1.967	0.224

Metabolites	FC	Direct	pvalu	VIP	qvalu
DN-isobutylamide	3.287	up	0.005	1.611	0.225
Narcimarkine	3.471	up	0.005	1.642	0.225
Thr Leu Ile	2.880	up	0.006	1.505	0.236
Heptadecanoic acid	0.323	down	0.006	2.231	0.721
Glucose pyruvate acetate	5.582	up	0.006	2.505	0.721
14-Dehydroergosterol	0.389	down	0.006	2.080	0.245
Isoleucyl-Threonine	3.230	up	0.006	1.649	0.245
alpha-Solanine	4.220	up	0.007	1.713	0.250
Melamine	17.111	up	0.007	1.694	0.252
Karanjin	4.348	up	0.007	1.721	0.263
L-Alanyl-L-leucine	2.583	up	0.008	1.538	0.265
CPA(18:0/0:0)	3.091	up	0.008	1.337	0.267
1alpha,2alpha,3beta,30-Tetrahydroxyurs-12-ene	0.370	down	0.009	1.907	0.272
Neurotensin 11-13	5.233	up	0.010	1.497	0.272
Glu Ile Leu	2.732	up	0.010	2.219	0.869
Decaethylene glycol	4.658	up	0.010	1.715	0.272
Longirabdolide F	0.080	down	0.010	1.703	0.272
2-Amino-5-chlorobenzoxazole	16.71	up	0.0111	1.768	0.272
Leu Ala Ile	2.686	up	0.011	2.296	0.869
Phe Pro Trp	4.105	up	0.011	1.524	0.272
N-(3-(Dimethylamino)propyl)acrylamide	2.649	up	0.011	1.456	0.272
N-METHYLCYCLOHEXYLAMINE	3.506	up	0.011	1.522	0.272
N-Docosahexaenoyl GABA	0.358	down	0.011	2.369	0.869
Lanceolitol A1	5.896	up	0.012	2.452	0.869
Histidylmethionine	2.669	up	0.012	1.477	0.277
8-Methyl-8-azabicyclo[3.2.1]oct-3-yl	3.837	up	0.012	1.698	0.277
Rigidiusculamide B	2.650	up	0.012	2.345	0.869
24-Methylenepollinastanone	0.367	down	0.012	2.452	0.869
Daumone 4	2.513	up	0.012	1.292	0.277
2-Hydroxydocosanoic acid	0.301	down	0.012	2.140	0.869
H-ILE-ILE-OH	2.581	up	0.013	1.424	0.277
norfentanyl	2.676	up	0.014	1.569	0.281
His His	2.898	up	0.014	1.376	0.281
2-Oleoyl-sn-glycero-3-phosphocholine	0.307	down	0.014	1.550	0.281
Lys Leu	2.514	up	0.014	1.462	0.281
Fomajorin S	21.32	up	0.014	1.965	0.281
oxametacin	6.819	up	0.015	2.035	0.932
Wilforlide A	0.351	down	0.015	1.340	0.281
Eriodictyol	0.126	down	0.017	2.174	0.932
N-Lauroyl Tryptophan	2.621	up	0.017	2.098	0.932
3-Hydroxy-4-methylanthranilate	32.75	up	0.017	1.536	0.299

Metabolites	FC	Direct	pvalu	VIP	qvalu
11,12-Methylenedioxykopsinaline	6.350	up	0.017	1.541	0.302
3-hydroxydecanoylcarnitine	2.848	up	0.018	1.392	0.303
rel-(-)-(1R,4R,10R)-4-methoxycembra-2E,7E,11	0.341	down	0.018	1.388	0.303
Leu Val Val	2.861	up	0.020	1.313	0.318
9(Z),11(E),13(E)-Octadecatrienoic Acid methyl	0.273	down	0.022	1.556	0.342
Brucine	3.812	up	0.024	1.391	0.356
Leu Ile Thr	2.951	up	0.025	1.969	0.983
FA 18:4+2O	0.362	down	0.026	2.146	0.983
Cardivin B	2.992	up	0.027	1.988	0.998
Leu-Phe-Asn	2.645	up	0.028	1.186	0.373
Isometheptene	2.700	up	0.028	1.342	0.373
1,4-Dideoxy-1,4-imino-D-arabinitol	3.051	up	0.029	1.430	0.373
Cichorioside L	2.588	up	0.029	1.849	0.998
Cardenolide	2.503	up	0.031	1.893	0.998
Nigellidine	4.222	up	0.032	1.154	0.387
Cypridinid etioluciferin	3.285	up	0.033	1.920	0.998
Valylhistidine	16.39	up	0.033	1.340	0.387
gamma-Tocotrienol	0.362	down	0.034	1.234	0.387
Lysylphenylalanine	2.729	up	0.034	1.261	0.387
DG(i-12:0/12:0/0:0)	18.47	up	0.035	1.530	0.390
MG(0:0/19:0/0:0)	0.357	down	0.035	1.149	0.390
Guanidinoethyl disulfide	19.59	up	0.035	1.291	0.391
D-Lysopine	2.567	up	0.036	1.241	0.393
Butanoic acid,	0.380	down	0.037	2.278	0.998
Stearoyllactic acid	0.233	down	0.037	1.970	0.998
omega-Cyclohexylundecanoic acid	0.173	down	0.037	1.885	0.998
senecionan	0.015	down	0.039	1.288	0.403
Celacinnine	4.553	up	0.039	1.234	0.403
Arg Leu	2.780	up	0.040	1.198	0.407
Robustamine cis-N-oxide	3.332	up	0.041	1.300	0.407
12,13-Deoxyroridin E	3.015	up	0.044	1.397	0.419
Lamiol	14.81	up	0.045	1.922	0.998
2,4,8-Eicosatrienoic acid isobutylamide	0.237	down	0.045	1.293	0.419
Arnidiol	0.120	down	0.046	1.356	0.419
Digitoxin	3.672	up	0.047	1.270	0.424
4-Methoxyphenol sulfate	3.410	up	0.047	1.799	0.998
Isotaifine	3.454	up	0.047	1.256	0.424
N-Oleoyl Histidine	0.392	down	0.048	1.902	0.998
Simmondsin	2.656	up	0.048	1.814	0.998
Deoxytryptoquivaline	0.221	down	0.049	1.625	0.998
